# Design exploration and comparative analysis of tail shape of tri-wheel-based stair-climbing robotic platform

**DOI:** 10.1038/s41598-022-24179-5

**Published:** 2022-11-14

**Authors:** JeongPil Shin, DongHan Son, YoungHwan Kim, TaeWon Seo

**Affiliations:** Department of Mechanical Convergence Engineering, Hanyang University, Seoul, 04763 Republic of Korea

**Keywords:** Engineering, Mechanical engineering

## Abstract

Stair climbing is one of the most important capabilities of mobile robots. Therefore, stair-climbing mobile robots have become a field of study and diverse stair-climbing mobile robots have been developed. Although tri-wheel-based stair-climbing robotic platforms were developed to overcome the challenges posed by stair climbing, they have shown limitations such as impact during locomotion and damage owing to friction with the nosing of the stairs. In this study, several tail mechanisms were proposed and designed to solve the limitations of tri-wheel-based stair-climbing robots. A comparative analysis of the tail mechanisms was performed through dynamic simulations based on various performance indices. It was observed that the tail mechanism improved the stability and stair-climbing performance of the tri-wheel-based stair-climbing robots. The experimental verification confirmed the reliability of the comparative analysis results based on the simulation. These findings can be used to design mobile stair-climbing robots.

## Introduction

In a future society where robots and humans coexist and cooperate, robots must be able to move harmoniously and smoothly in diverse human environments. Stairs are among the most insurmountable obstacles encountered by robots in indoor environments. However, stair climbing is an indispensable capability for indoor service robots. Therefore, several robotic platforms have been developed to facilitate this capability. SpotMini, a quadrupedal robot developed by Boston Dynamics, can climb stairs stably and quickly. Legged robots, such as quadrupedal and bipedal robots, can overcome complex ground conditions, such as narrow passages or stairs; however, their postural control and mechanisms are quite complicated. Furthermore, because legged robots have some weaknesses, robots with simple mechanisms, such as wheels, tracks, and linkages, have been proposed. Tracked robots, which embody the most common mechanisms for overcoming obstacles, require simple control systems and designs^1,2^. As a wheel-linkage mechanism, the eccentric crank rover, a novel crank wheel mechanism with eccentric wheels and four-bar linkages, achieved high efficiency and mobility in rough terrain^3^. One of the robots with various mechanisms to overcome challenges related to stair climbing is a tri-wheel-based robot.

Several studies have been conducted on tri-wheel-based robotic platforms for stair climbing. Several of the proposed tri-wheel-based stair-climbing robots are listed in Table 1. A curved-spoke tri-wheel (CSTW) mechanism, which is a tri-wheel mechanism with a curved spoke and stopper mechanism, has been used to achieve fast and robust stair climbing^4^. Tri-Wheel, a spoke drive mechanism capable of two rotational modes, driving and tumbling modes, can drive quickly on smooth surfaces on high speeds and climb tall objects^5^. STEP, a mobile platform with 2-DOF transformable wheels, has been used to successfully climb steps and stairs of various sizes encountered in indoor environments^6^. LEVO, a mobile robotic platform using wheel-mode switching primitives, has been proposed for stair-climbing and high-moving performance on flat ground^7^. Tri-wheel-based systems have been extensively studied; however, these systems have several drawbacks and their effectiveness can be affected by factors such as impact during locomotion (CSTW) (LEVO), damage and friction (resistance) problems owing to contact with the nosing of the stairs (CSTW) (STEP) (LEVO), complicated design (STEP) (Tri-Wheel) (LEVO), and a decline in stair-climbing capability with payload (LEVO). These drawbacks degrade the performance of tri-wheel-based stair-climbing robotic platforms, specifically their stability and climbing ability.

**Table 1:** Comparison of various tri-wheel-based stair-climbing robots.

Name	CSTW	Tri-wheel	STEP	LEVO
Picture				
Mechanism	Curved-spoke tri-wheel	Tri-Wheel	2-DOF transformable wheel	CSTW with switching primitives
Flat terrain mobility	−	+	−	+
Stair-climbing stability	−	+	−	−
Design complexity	+	−	−	−
Characteristic	Fast climbing speed	Smooth and rapid level ground operation Tumbling mode	Shape-morphing wheel	Wheel-mode switching Flat ground operation

In this study, several tail mechanisms were devised and proposed to solve the aforementioned limitations of tri-wheel-based stair climbing robots, specifically as pertains to impact during locomotion and damage and friction problems owing to contact with the nosing. Furthermore, this study sought to create tail mechanisms that could improve stair-climbing performance while being simple and passive without applying an actuator or complex mechanism, and that could also enable flat driving. As a tri-wheel-based stair-climbing robotic platform, LEVO with a normal wheel and a CSTW was used.

Quantitative and qualitative comparisons were carried out to evaluate the proposed tail mechanisms for tri-wheel-based robots through dynamic simulation using a commercial dynamic simulation tool (software) based on various performance indices, mechanical complexity, center of mass (CM) trajectory, acceleration of CM, friction requirement, torque requirement, and climbing speed. The stair-climbing performance and stability of the proposed tail mechanisms was compared and analyzed through the simulation for each case of the tail mechanism, and the case, which had the smallest minimum required friction coefficient acting between the CSTW and surface of the stairs, of each tail mechanism was selected. However, the minimum required friction coefficient is a value that cannot be obtained through the dynamic equation and can be obtained through the static analysis. And, the static analysis cannot obtain the minimum required friction coefficient by reflecting the climbing speed, which was 1.0 step/s (20 rpm). Thus, in this study, the stair-climbing simulations were performed using a commercial dynamic simulation tool (software), RecurDyn, instead of creating the dynamic simulation model by deriving kinematic or dynamic equations of each tail mechanism.

Tails are of significant interest to robotic engineers and biologists. They are an indispensable apparatus for climbing robots and other creatures. There are some abilities that are availed by tails while climbing such as holding onto supports, maintaining balance, and moving from one place to another^8^. Further, tails grant animals and robots postural control and increased stability^9^. Treecreepers, like wood peckers, use their tails for support during climbing, a habit associated with structural adaptations different from those involved in trunk-climbing without tail-support^10^. The gecko, a reptile that exhibits arboreal acrobatics, utilizes its tail not only as an emergency fifth leg to prevent falling during rapid climbing but also as a highly active control appendage^11^.

Tail mechanisms have also been studied by many researchers in robotics for postural control to avoid sudden tilting or falls. A miniature two-wheg climbing robot that incorporates a simple passive tail, while maintaining its compactness, was proposed to design a tail with such a shape that minimizes certain requirements for external transitioning^12^. Curve fitting was performed to mimic the curve of a tail, minimizing the adhesive requirements and enhancing not only the payload but also the speed capabilities of the climbing robot^13^. Combot^14^, a compliant climbing robotic platform with transitioning capability and high payload capacity, can perform internal and external transitions using compliant torques from torsion springs and an active tail. Climbing robots are prone to falls because of the pitch-back moment; therefore, the active tail compensates the pitch-back moment and enables stability by mechanically supporting the robot when it performs external transitions against the direction of gravity.

**Figure 1:**
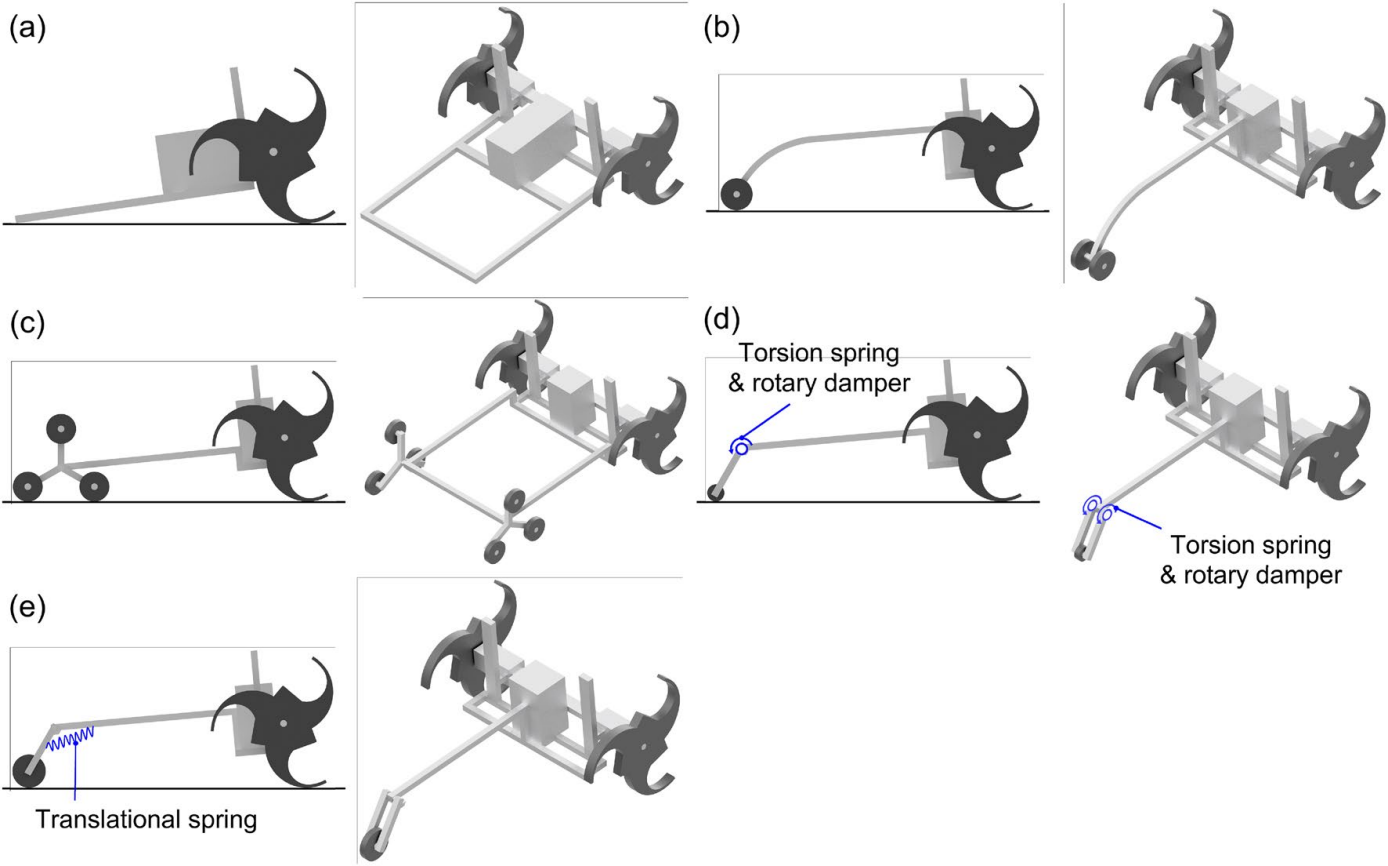
Simple models of the five tail mechanisms: (**a**) Basic tail mechanism (DOF1=0, DOF2=2), (**b**) Curved linkage mechanism (DOF1 = 2, DOF2 = 4), (**c**) Tri-wheel mechanism (DOF1 = 8, DOF2 = 10), (**d**) Compliant mechanism with torsion spring and rotary damper (CPL-SD) (DOF1 = 3, DOF2 = 5), and (**e**) Compliant mechanism with translational spring (CPL-S) (DOF1 = 4, DOF2 = 6). Degree of freedom of the tail mechanism is presented for the case of considering only tail mechanism parts (DOF1) and the case of considering the whole robotic system (DOF2). Software used for this figure: Microsoft PowerPoint Microsoft 365 MSO (Version 2209 Build 16.0.15629.20200) 64-Bit (https://www.microsoft.com/en-us/microsoft-365/powerpoint), Autodesk Inventor 2023.1.1 (https://www.autodesk.com/products/inventor/overview?term=1-YEAR &tab=subscription).

In this study, five different types of tail mechanisms, including the basic tail mechanism^7^ for a tri-wheel-based stair-climbing robot, were proposed and compared. The simple models of the five tail mechanisms considered in the dynamic simulation are presented in Fig. 1. The five tail mechanisms are the basic tail mechanism, curved linkage mechanism, tri-wheel mechanism, compliant mechanism with torsion spring and rotary damper (CPL-SD) and compliant mechanism with translational spring (CPL-S). It was found that the proposed tail mechanisms enable tri-wheel-based stair-climbing robots maneuver passively during stair-climbing without colliding with the nosing. Thus, the findings of this study can be exploited for designing mobile robots for stair-climbing applications.

The remainder of this paper is organized as follows. The design proposal for the various tail mechanisms is described in “Tail mechanism design”. In “Comparison with dynamic simulation”, the performance indices for comparing the five tail mechanisms and the dynamic simulation environment are presented, and the comparison results of the dynamic simulation of the tail mechanisms are described. In “Experimental verification and results”, experimental verification was carried out and it confirmed the reliability of the comparative analysis results based on the dynamic simulation. Finally, in “Conclusion”, concluding remarks and future work are presented.

## Tail mechanism design

In this study, the design of the tail mechanism was made through the setting of several design variables for each tail mechanism. For each type of tail mechanism, the case with the smallest minimum required friction coefficient acting between the CSTW and the stair surface was selected using dynamic simulation. And, as will be explained in detail in “Comparison with dynamic simulation”, the tail mechanisms proposed in this study were compared using various performance indices. One of the various performance indices is the minimum required friction coefficient used in the design of the tail mechanism. The coefficient of friction is very important in evaluating a mobile robot that climbs stairs. The reason is that, in order for the mobile robot to climb stairs stably, frictional force between the driving wheel and the stair surface must be sufficiently generated so that slip does not occur, and this frictional force is determined by the friction coefficient. Therefore, in the performance evaluation of the stair climbing robot, the stair climbing performance and stability can be evaluated using the minimum required friction coefficient required to climb stairs stably without slipping.

### Basic tail mechanism

**Figure 2:**
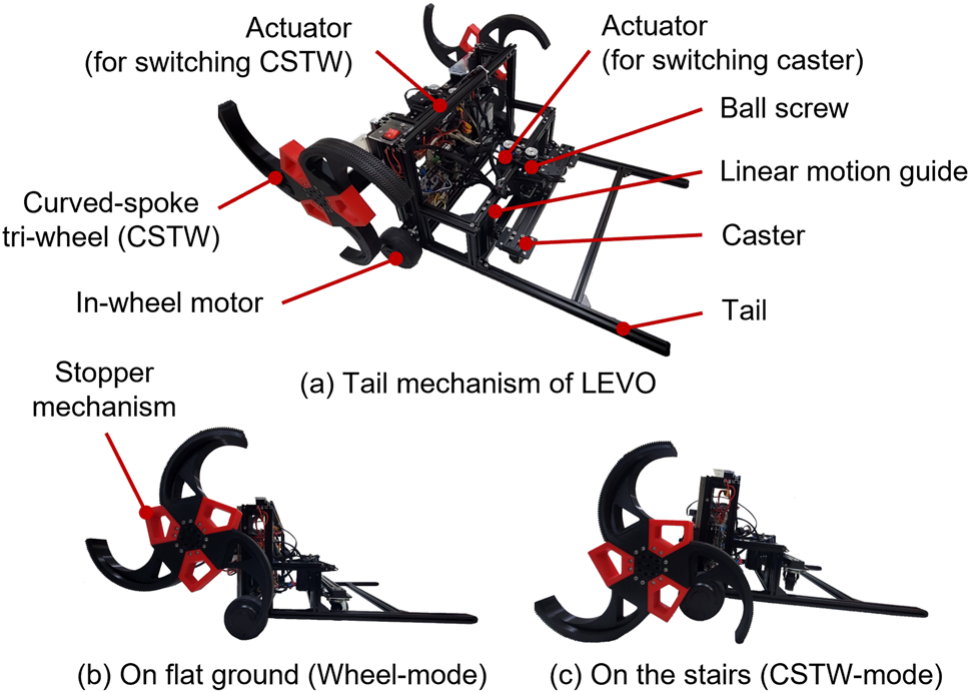
Basic tail mechanism. Software used for this figure: Microsoft PowerPoint Microsoft 365 MSO (Version 2209 Build 16.0.15629.20200) 64-Bit (https://www.microsoft.com/en-us/microsoft-365/powerpoint).

The basic tail mechanism, the tail mechanism of LEVO^7^, was used as a conventional tail mechanism^4,6,8,15–19^. LEVO utilizes a normal wheel for driving on flat terrains and a curved-spoke tri-wheel (CSTW) for climbing stairs, as shown in Fig. 2. To operate both mechanisms independently, a switching mechanism with ball screws, linear motion guides, and actuators was adopted. LEVO has two driving modes: wheel mode using two in-wheel motors and two casters for driving on flat ground and CSTW mode using two curved-spoke tri-wheels (CSTW) and a tail for climbing stairs.

**Figure 3:**
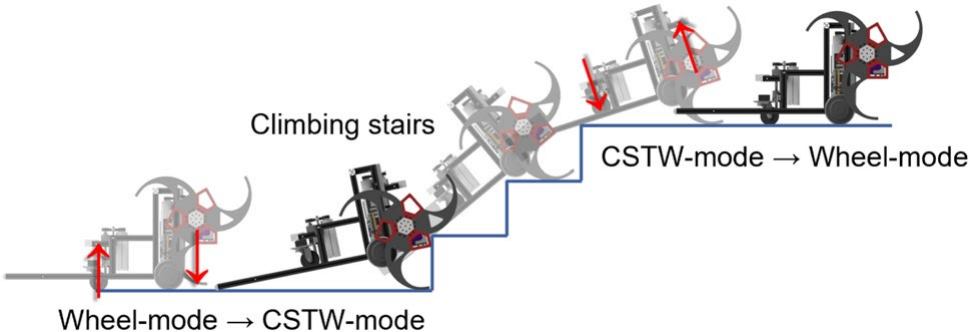
Stair-climbing process of LEVO. Software used for this figure: Microsoft PowerPoint Microsoft 365 MSO (Version 2209 Build 16.0.15629.20200) 64-Bit (https://www.microsoft.com/en-us/microsoft-365/powerpoint), Autodesk Inventor 2023.1.1 (https://www.autodesk.com/products/inventor/overview?term=1-YEAR &tab=subscription).

The basic tail mechanism is simple. It only consists of a few aluminum frames and it helps the robot to maintain four-point support (two-point support by two CSTWs and two-point support by the tail) when the robot climbs the stairs. The stair-climbing process for the LEVO is shown in Fig. 3. When the robot encounters the stair, the CSTW mode is initiated such that the curved-spoke tri-wheels are lowered and the caster in the rear is lifted. Consequently, the curved-spoke tri-wheel and tail contact the ground and the robot climbs the stairs by rotating its CSTW, with the tail maintaining the two-point support.

However, the tail of LEVO has several drawbacks: damage and friction (resistance) problems owing to contact with the nosing and impact during locomotion. These shortcomings reduce the stability and stair-climbing capability of the robot. The friction problem, in particular, significantly affects the stair-climbing performance of the LEVO. As can be seen in Fig. 8a, LEVO with its tail mechanism can climb $300 \times 160$ mm stairs without slipping. Nevertheless, this is possible only when the coefficient of friction between the nosing and basic tail mechanism is 0.1. Therefore, when the coefficient of friction increased, the stair-climbing performance was severely degraded.

### Curved linkage mechanism

A curved linkage mechanism was proposed as a simple tail mechanism for passive stair climbing. The curved linkage mechanism was a tail mechanism with a curved tail to prevent collisions and friction with the nosing, which are limitations of tri-wheel-based stair-climbing robots with a conventional tail mechanism such as the basic tail mechanism.

**Figure 4:**
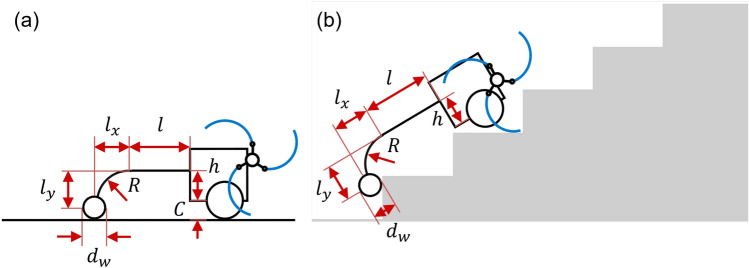
Design parameters of the curved linkage mechanism: (**a**) on flat ground, (**b**) on the stairs. Software used for this figure: Microsoft PowerPoint Microsoft 365 MSO (Version 2209 Build 16.0.15629.20200) 64-Bit (https://www.microsoft.com/en-us/microsoft-365/powerpoint).

The design parameters of the curved linkage mechanism are shown in Fig. 4. The design parameters of the curved linkage mechanism were *l*, $l_x$, $l_y$, $d_w$, *h*, and *R*. *l*, $l_x$, and $l_y$ denote the horizontal linkage length, *x*-directional curved linkage length, and *y*-directional curved linkage length, respectively. $d_w$, *h*, and *R* indicate the diameter of a wheel, the height of the fastening position where the tail mechanism is connected to the body, and the radius of curvature of the curved linkage, respectively. *C* is the distance between the ground and the base of the body, which is 38.5 mm.

The tri-wheel based robot with the curved linkage mechanism can not only climb stairs but also drive on flat ground. Therefore, as illustrated in Fig. 4a, the robot with the curved linkage mechanism should maintain level posture on the flat ground. The *y*-directional curved linkage length $l_y$, wheel diameter $d_w$, and height *h* have the following algebraic relations:

1 $$\begin{aligned} C+h=\frac{d_w}{2}+l_y. \end{aligned}$$

As shown in Table 2, the design parameters of the curved linkage mechanism can be classified into four cases based on the size of the wheel $d_w$. To prevent collision or friction owing to contact between the nosing and curved tail mechanism while climbing stairs with dimensions of $300 \times 160$ mm, the design parameters *l*, $l_x$, $l_y$, and *R* were selected for each case, as shown in Table 2. *h* was determined using the horizontal conditional expression in Eq. (1). When climbing stairs, the pitch-back moment acting on the robot increased when the horizontal linkage length *l* was small and the tilt angle of the robot was large, which impeded the performance of climbing stairs. If *h* becomes smaller (when $d_w$ is the same value), the position of center of mass of the robot gets lower and $l_y$ should be reduced by the relation of Eq. (1). A small $l_y$ led to interference and collisions between the curved linkage mechanism and nosing. In terms of $d_w$, the stair-climbing ability of a robot with a curved linkage mechanism was largely determined by the wheel size $d_w$. As the $d_w$ grows, a larger minimum required friction coefficient is required for stable stair-climbing without slipping. Therefore, the figures for each design parameter must be determined based on these conditions.

**Table 2:** Various design parameters used in the dynamic simulation of the curved linkage mechanism.

Design parameter	*l* (mm)	$l_x$ (mm)	$l_y$ (mm)	*R* (mm)	$d_w$ (mm)	*h* (mm)
Case 1	350	164	150	164.7	50	136.5
Case 2	400	250	150	283.3	100	161.5
Case 3	300	214	150	250	150	186.5
Case 4	300	214	100	250	200	161.5

The stair-climbing performance was verified through dynamic simulation for each case, and Case 2, which had the smallest minimum required friction coefficient acting between the CSTW and surface of the stairs, was selected. For Case 2 of the curved linkage mechanism, when the coefficient of friction was 0.60, the tri-wheel-based robot with the curved linkage mechanism could climb stairs without slipping. As can be seen in Fig. 8b, the tri-wheel based robot with the curved linkage mechanism (Case 2) effectively climbed the $300 \times 160$ mm stairs without slipping or colliding with the nosing.

### Tri-wheel mechanism

A tri-wheel mechanism that is often adopted because it enables passive stair-climbing was proposed as a tail mechanism. The mechanism not only enabled stable stair-climbing but also prevented collisions and friction problems with the nosing. Because the mechanism conferred the dual capacity of rolling and climbing, it enables stair-climbing robots to climb stairs by rotating around their central axle and rolling along the surface of the stair until they reached a perfect position to flip over and continue the climbing process^5^.

**Figure 5:**
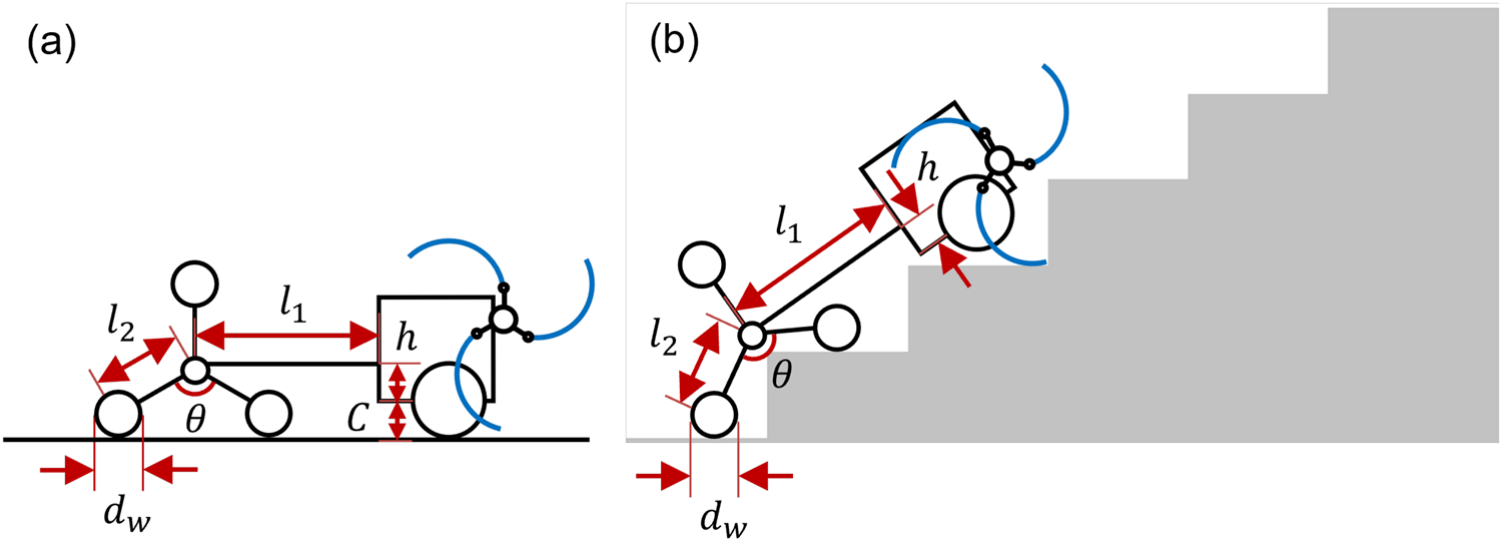
Design parameters of the tri-wheel mechanism: (**a**) on the flat ground, (**b**) on the stairs. Software used for this figure: Microsoft PowerPoint Microsoft 365 MSO (Version 2209 Build 16.0.15629.20200) 64-Bit (https://www.microsoft.com/en-us/microsoft-365/powerpoint).

The design parameters of the tri-wheel mechanism are presented in Fig. 5. The design parameters of the tri-wheel mechanism are $l_1$, $l_2$, $\theta$, $d_w$, and *h*. $l_1$ and $l_2$ represent the horizontal linkage length and radius length of the tri-wheel, respectively. $d_w$ and *h* refer to the diameter of a wheel and height of the fastening position where the tail mechanism is connected to the body, respectively. In this study, $\theta$ was set as 120$^{\circ }$ to create a tri-wheel mechanism. *C* is the distance between the ground and base of the body, which is 38.5 mm.

Stair-climbing robots with the tri-wheel mechanism can not only climb stairs but can also maneuver on flat ground. Therefore, as illustrated in Fig. 5a, these robots should maintain level posture on flat ground. The radius of the tri-wheel $l_2$, wheel diameter $d_w$, and height *h* have the following algebraic relation:

2 $$\begin{aligned} C+h=\frac{d_w}{2}+l_2cos\frac{\theta }{2}. \end{aligned}$$

Furthermore, for a stable stair-climbing capability, the radius length of the tri-wheel $l_2$ and wheel diameter $d_w$ of the tri-wheel should satisfy the following inequality constraints:

3 $$\begin{aligned}{} & {} l_2+\frac{d_w}{2}\ge h_s, \end{aligned}$$

4 $$\begin{aligned}{} & {} d_w+2l_2sin\frac{\theta }{2}\le w_s, \end{aligned}$$

5 $$\begin{aligned}{} & {} \therefore h_s-\frac{d_w}{2}\le l_2\le \frac{w_s-d_w}{2sin\frac{\theta }{2}}, \end{aligned}$$

where $h_s$ is the stair riser height (160 mm) and $w_s$ is the stair nosing depth (300 mm).

As shown in Table 3, the design parameters of the tri-wheel mechanism were classified into five cases according to the size of the wheel $d_w$. The design parameters $l_1$, $l_2$, and $d_w$ in Table 3 were selected such as to ensure case had an excellent stair-climbing ability and to prevent the collision or friction caused by contact between the tri-wheel tail mechanism and the nosing when climbing stairs with dimensions of $300 \times 160$ mm. The design parameters $l_2$ and $d_w$ were determined using Inequal. (3), (4), and (5) (inequality constraints mentioned above). Furthermore, *h* is determined using the level conditional expression in Eq. (2). When the linkage length $l_1$ was small and the tilt angle of the robot was large during stair climbing, the pitch-back moment acting on the robot increased, resulting in a decrease in the stair-climbing performance. In the case of $d_w$, the stair-climbing ability of the robot with the tri-wheel mechanism was significantly affected by the wheel size $d_w$. As the $d_w$ grows, a larger minimum required friction coefficient is required for stable stair-climbing without slipping. Therefore, the values of each design parameter were determined based on these conditions.

**Table 3:** Cases of design parameters used in the dynamic simulation of the tri-wheel mechanism.

Design parameter	$l_1$ (mm)	$l_2$ (mm)	$\theta$ (°)	$d_w$ (mm)	*h* (mm)
Case 1	523	120	120	80	61.5
Case 2	550	115	120	90	64
Case 3	523	110	120	100	66.5
Case 4	523	105	120	110	69
Case 5	523	100	120	120	71.5

The stair-climbing performance was verified through dynamic simulation for each case, and Case 2, with the least minimum required friction coefficient acting between the CSTW and the surface of the stairs, was selected. For Case 2 of the tri-wheel mechanism, when the coefficient of friction was 0.50, the robot with the tri-wheel tail mechanism succeeded at climbing the stairs without slipping. As shown in Fig. 8c, it was confirmed that the robot with the tri-wheel mechanism (Case 2) successfully climbed $300 \times 160$ mm stairs without slipping or colliding with the nosing.

### CPL-SD

A CPL-SD is a tail mechanism composed of two linkages connected with a revolute joint, two torsion springs and two rotary dampers, and a stopper. It was designed to improve the stability and performance of a tri-wheel-based robots during stairs climbing. Since the curved linkage mechanism described in Subsection “Curved linkage mechanism” comprised of a stiff (rigid) structure without compliance, the degree of improvement in the stair-climbing performance and stability was small. To overcome the limitations of the curved linkage mechanism, a compliant mechanism with a torsion spring and rotary damper was devised.

**Figure 6:**
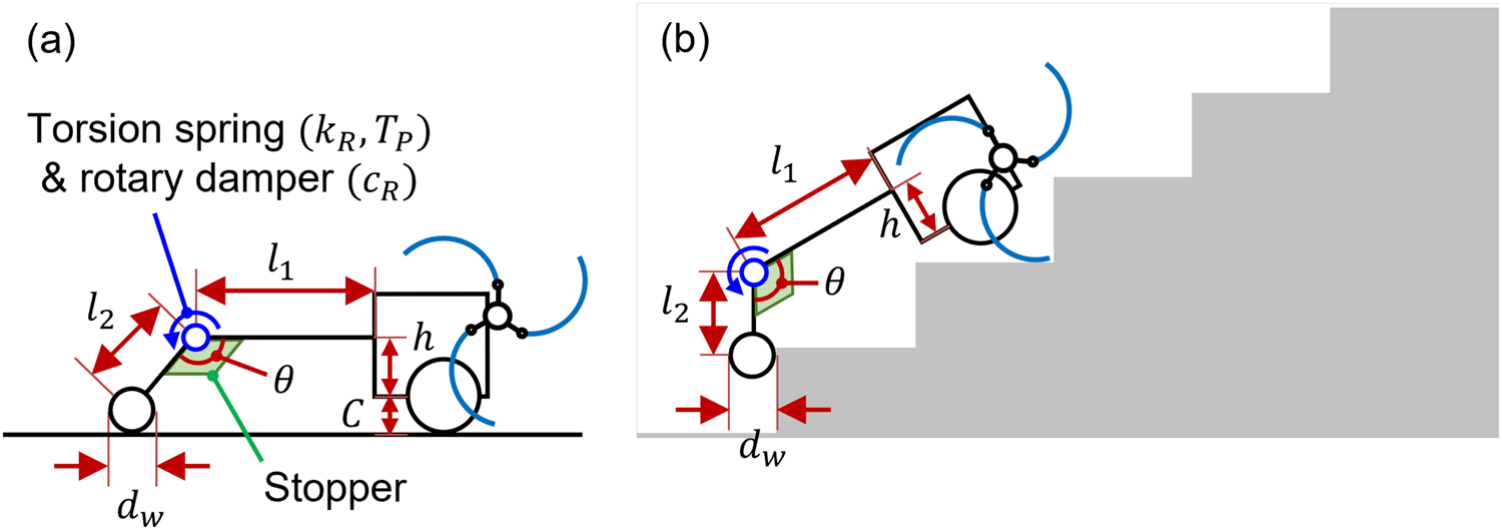
Design parameters of the CPL-SD: (**a**) on the flat ground, (**b**) on the stairs. Software used for this figure: Microsoft PowerPoint Microsoft 365 MSO (Version 2209 Build 16.0.15629.20200) 64-Bit (https://www.microsoft.com/en-us/microsoft-365/powerpoint).

Figure 6 presents the design parameters of the CPL-SD. The design parameters of the CPL-SD were $l_1$, $l_2$, *h*, $d_w$, $\theta$, $k_R$, $c_R$ and $T_P$. The design parameters $l_1$ and $l_2$ denote the linkage length, respectively. The design parameters *h* and $d_w$ refer to the height of the fastening position where the tail mechanism is connected to the body and the diameter of a wheel, respectively. The design parameters $\theta$, $k_R$, and $c_R$ indicate the angle between two linkages, the spring constant (spring stiffness) of the torsion spring, and the damping coefficient of the rotary damper, respectively. The design parameter $T_P$ represents the preload (pre-torque) of the torsion spring. *C* is the distance between the ground and base of the body, which is 38.5 *mm*.

A tri-wheel-based stair-climbing robot with a CPL-SD can climb stairs and travel on flat ground. Therefore, as illustrated in Fig. 6a, the robot with the CPL-SD should maintain level posture on the flat ground. Accordingly, the linkage length $l_2$, diameter of a wheel $d_w$, and height *h* have the following algebraic relation:

6 $$\begin{aligned} C+h=\frac{d_w}{2}+l_2cos(\theta -90^{\circ }). \end{aligned}$$

Furthermore, for the CPL-SD to possess stable stair-climbing ability, the radius length of the linkage length $l_2$ and the wheel diameter $d_w$ should satisfy the following inequality constraints:

7 $$\begin{aligned} l_2+\frac{d_w}{2}\ge h_s, \end{aligned}$$

where $h_s$ is the stair riser height (160 mm).

In Table 4, the design parameters of the compliant mechanism with the torsion spring and rotary damper are classified into four cases, according to the size of the wheel $d_w$. To prevent collision or friction by contact between the nosing and the CPL-SD when climbing stairs with dimensions of $300 \times 160$ mm, the design parameters of the CPL-SD ($l_1$, $l_2$, *h*, $d_w$, $\theta$, $k_R$, $c_R$, and $T_P$) were determined for each case shown in Table 4. The design parameters $l_2$ and $d_w$ were determined using Inequal. (7) (the inequality constraint stated above). *h* was determined using the horizontal conditional expression in Eq. (6). When ascending stairs, the small link length $l_1$ and large tilt angle of the robot increased the pitch-back moment acting on the robot, affecting the performance of climbing stairs. In terms of $d_w$, the stair-climbing capability of the tri-wheel-based robot with the CPL-SD was significantly affected by the wheel size $d_w$. As the $d_w$ grows, a larger minimum required friction coefficient is required for stable stair-climbing without slipping. Here, the angle between linkages $\theta$ was set at 125$^{\circ }$ to make the linkage of length $l_2$ meet the riser of the stair almost vertically. Because the linkage of the length $l_2$ came into contact with the riser of the stair vertically, the angle of deformation of the torsion spring was small; therefore, the reaction torque caused by the torsion spring decreased. If the reaction torque was large, the curved-spoke tri-wheel, which is the driving wheel of the robot, slipped. In addition, to reduce the angle of deformation of the torsion spring, for the spring constant (spring stiffness), a torsion spring $k_R$ was selected among the available and suitable spring constants within the existing spring constant range of the torsion spring. However, as the spring constant $k_R$ increased, the torsion spring collided with the stopper and vibrated as it recovered from the deformation. The stability evaluation indicators used in this study indicated that the stability of the stair-climbing robot was affected by this vibration generation. Therefore, a rotary damper was adopted to decrease vibration. Its damping coefficient $c_R$ was selected among the damping constant values of the actual rotary damper to sufficiently suppress the occurrence of these vibrations and ensure stable stair-climbing. Moreover, even if a large spring constant value $k_R$ was selected from the existing torsion spring constant range, the deformation angle of the torsion spring when the wheel touched the stairs was significant, which increased the reaction torque caused by the torsion spring and affected the stair-climbing performance. The preload (pre-torque) of the torsion spring $T_P$ was used to solve this problem. Therefore, the figure for each design parameter was determined based on these conditions.

**Table 4:** Cases of design parameters used in the dynamic simulation of the CPL-SD.

Design parameter	$l_1$ (mm)	$l_2$ (mm)	*h* (mm)	$d_w$ (mm)	$\theta$ ($^{\circ }$)	$k_R$ (N mm/$^{\circ }$)	$c_R$ (N mm s/$^{\circ }$)	$T_P$ (N mm)
Case 1	540	150	109.4	50	125	100	FDN-70A-L/R114 Rotary damper	1500
Case 2	540	150	134.4	100	125	100	FDN-70A-L/R114 Rotary damper	1500
Case 3	540	200	190.3	150	125	100	2000	3500
Case 4	540	200	200.3	200	125	100	2500	4500

The stair-climbing performance was verified through a dynamic simulation for each case, and Case 1 with the least minimum required friction coefficient acting between the CSTW and the surface of the stairs was selected. For Case 1 of the CPL-SD, when the coefficient of friction was 0.47, the tri-wheel-based robot with the CPL-SD successfully climbed the stairs without slipping. As shown in Fig. 8d, it was confirmed that the tri-wheel based robot with the CPL-SD (Case 1) could climb the $300 \times 160$ mm stairs without slipping or colliding with the nosing.

### CPL-S

**Figure 7:**
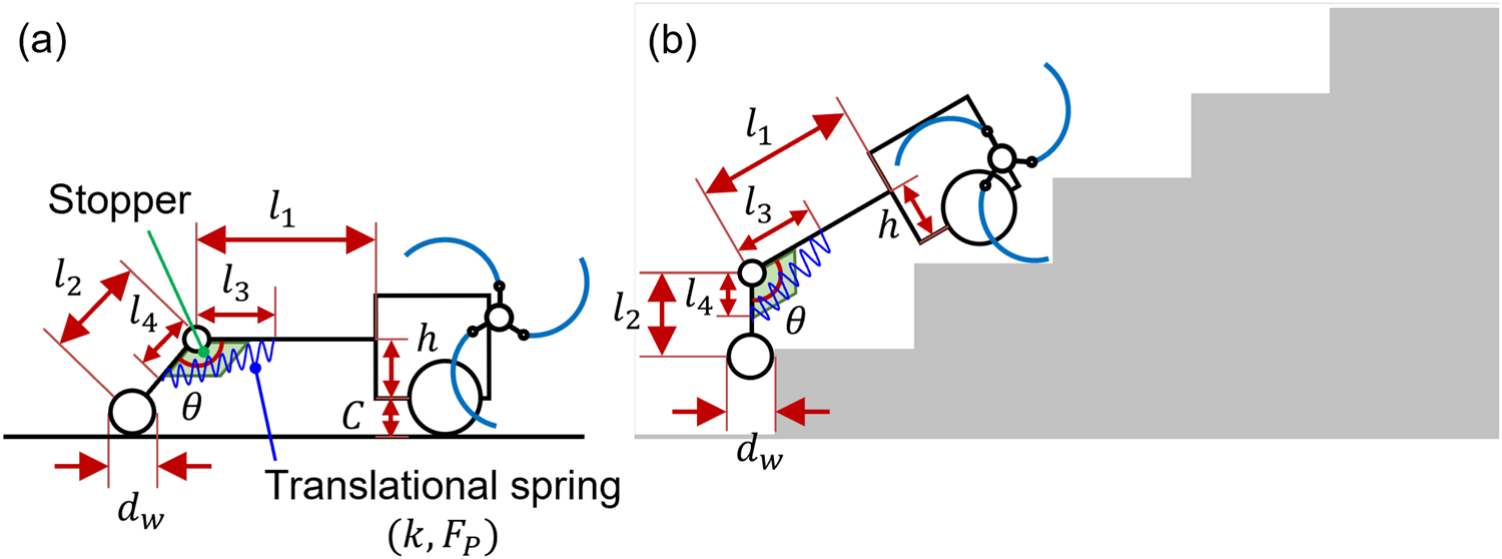
Design parameters of the CPL-S: (**a**) on the flat ground, (**b**) on the stairs. Software used for this figure: Microsoft PowerPoint Microsoft 365 MSO (Version 2209 Build 16.0.15629.20200) 64-Bit (https://www.microsoft.com/en-us/microsoft-365/powerpoint).

A CPL-S is a tail mechanism with a translational spring at the tail composed of two linkages and a stopper. As shown in Fig. 7, two linkages are connected with a revolute joint and a translational spring that makes resilience that allows sufficient traction force to develop between the wheel and the stair riser when the wheel comes into contact with the stair riser. Similar to the CPL-SD, the compliant mechanism with a translational spring was designed to overcome the limitations of the curved linkage mechanism and improve the stability and performance of the tri-wheel-based stair-climbing robot through the compliance of the translational spring. However, the CPL-SD also has a limitation in that it has a torsion spring with low spring stiffness. Therefore, the CPL-S, a compliant mechanism with a translational spring with high spring stiffness, was devised.

Figure 7 shows the design parameters of the CPL-S. The design parameters of the CPL-S are $l_1$, $l_2$, $l_3$, $l_4$, *h*, $d_w$, $\theta$, *k* and $F_P$. $l_1$ and $l_2$ denote the linkage length. $l_3$ and $l_4$ are the lengths from the joint to anchoring points of the translational spring. *h*, $d_w$, and $\theta$ represented the height of the fastening position where the tail mechanism is connected to the body, the diameter of a wheel, and the angle between two linkages, respectively. *k* and $F_P$ represent the spring constant (spring stiffness) of the translational spring and the preload of the translational spring, respectively. *C* is the distance between the ground and base of the body, which is 38.5 *mm*.

The tri-wheel-based stair-climbing robot with a CPL-S can climb stairs and travel on flat ground. Accordingly, as illustrated in Fig. 7a, the robot with the CPL-S should maintain level posture on the flat ground. Consequently, the linkage length $l_2$, diameter of the wheel $d_w$, and height *h* had the following algebraic relation:

8 $$\begin{aligned} C+h=\frac{d_w}{2}+l_2cos(\theta -90^{\circ }). \end{aligned}$$

In addition, to ensure that the CPL-S was capable of stable stair climbing, the radius length of the tri-wheel $l_2$ and wheel diameter $d_w$ should satisfy the following inequality constraints:

9 $$\begin{aligned} l_2+\frac{d_w}{2}\ge h_s, \end{aligned}$$

where $h_s$ is the stair riser height (160 mm).

As shown in Table 5, the design parameters of the CPL-S are classified into four cases according to the size of the wheel $$d_w$$. To prevent collision or friction by contact between the edge of the stairs and the CPL-S when climbing stairs with dimensions of $$300 \times 160$$ mm, the design parameters of the CPL-S ($$l_1$$, $$l_2$$, $$l_3$$, $$l_4$$, *h*, $$d_w$$, $$\theta $$, *k*, and $$F_P$$) were determined for each case, as shown in Table 5. The design parameters $$l_2$$ and $$d_w$$ were determined using Inequal. (9) (the inequality constraint mentioned above). Furthermore, *h* was determined using the horizontal conditional expression in Eq. (8). If the linkage length $$l_1$$ was small and the tilt angle of the robot was large during stairs climbing, the pitch-back moment acting on the robot increased, resulting in a decrease in the stair-climbing performance. For wheel size $$d_w$$, the climbing capability of the tri-wheel-based robot with the CPL-S was primarily determined by the size of the wheel $$d_w$$. As the $$d_w$$ grows, a larger minimum required friction coefficient is required for stable stair-climbing without slipping. The angle between linkages $$\theta $$ was set at 125$$^{\circ }$$ to enable the linkage of length $$l_2$$ contact the riser of the stair almost vertically. Because the linkage of length $$l_2$$ came into contact with the riser of the stair vertically, the collision or friction between the CPL-S tail mechanism and stairs was circumvented when the spring constant (spring stiffness) of the translational spring *k* was sufficiently large. However, if *k* was very large, the spring was unable to absorb the resultant impact when the wheel touched the stairs and acted like a rigid structure such as a curved linkage mechanism. Accordingly, the preload of the translational spring $$ T_P$$ was adopted for the CPL-S to determine the appropriate *k* value (which was not very large). Therefore, the figure for each design parameter was determined based on these considerations.

**Table 5:** Cases of design parameters used in the dynamic simulation of the compliant mechanism with translational spring.

Design parameter	$l_1$ (mm)	$l_2$ (mm)	$l_3$ (mm)	$l_4$ (mm)	*h* (mm)	$d_w$ (mm)	$\theta$ (°)	*k* (N/mm)	$F_P$ (N)
Case 1	540	150	135	75	109.4	50	125	20	200
Case 2	540	150	135	75	134.4	100	125	30	300
Case 3	540	200	150	80	190.3	150	125	30	300
Case 4	540	200	150	80	200.3	200	125	-	-

**Figure 8:**
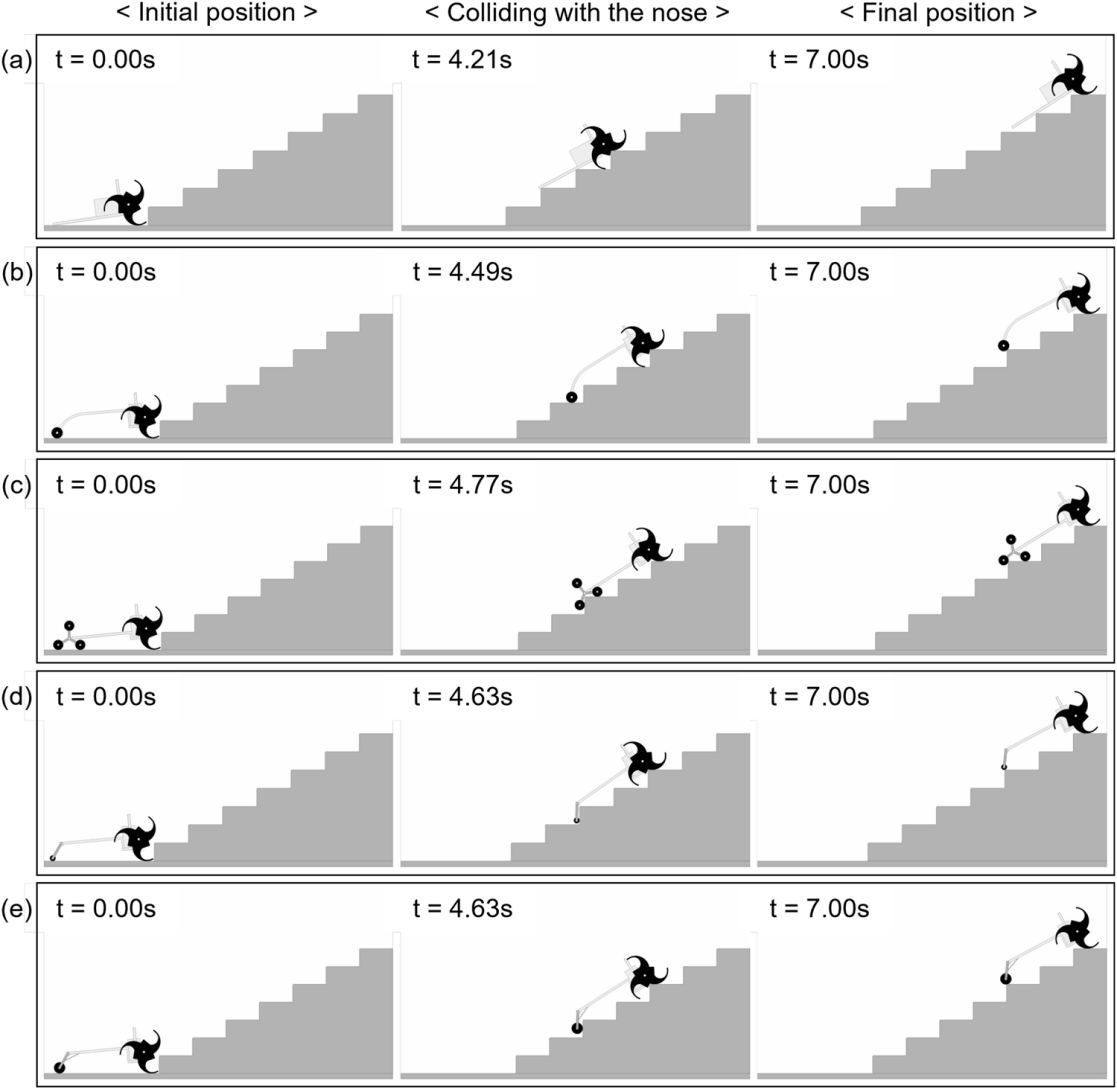
Ability of each designed tail mechanism to facilitate the ascension of $300 \times 160$ mm stairs without slipping was confirmed through simulation. (**a**) Basic tail mechanism, (**b**) Case 2 of curved linkage mechanism, (**c**) Case 2 of tri-wheel mechanism, (**d**) Case 1 of CPL-SD, and (**e**) Case 2 of CPL-S. Software used for this figure: Microsoft PowerPoint Microsoft 365 MSO (Version 2209 Build 16.0.15629.20200) 64-Bit (https://www.microsoft.com/en-us/microsoft-365/powerpoint), RecurDyn V9R1 (9.1.23284.0.9108.3) (https://support.functionbay.com/en/page/single/2/recurdyn-overview).

The stair-climbing performance was verified through dynamic simulation for each case, and Case 2, with the smallest minimum required friction coefficient acting between the CSTW and the surface of the stairs, was chosen. For Case 2 of the CPL-S, when the coefficient of friction was 0.51, the tri-wheel-based robot with the CPL-S successfully climbed the stairs without slipping. As shown in Fig. 8e, the tri-wheel based robot with the CPL-S case 2 successfully ascended the $300 \times 160$ mm stairs without slipping and friction or collision with the nosing.

## Comparison with dynamic simulation

### Performance indices

There are a variety of performance evaluation indices for comparing the performance of different mechanisms. Nie^20^ proposed an indicator called mechanical complexity to compare the complexities of robots. Thueer^21,22^ proposed several performance indices, such as friction requirement and slip and torque requirements, to comparatively analyze mobile robots. In this study, the root mean square (RMS) translational and angular accelerations and RMS driving torque were proposed to determine the stability and stair-climbing performance. In addition, the trajectory of the CM, which is commonly used for the performance evaluation of a robot, and the climbing speed, one of the most perceptible and simplest performance evaluation indices for different stair-climbing mechanisms, were also proposed.

#### Mechanical complexity

In this study, mechanical complexity, a relatively simple metric, was used to evaluate and compare the complexity of robots with different tail mechanisms. The mechanical complexity was assumed to be related to the number of actuators and the order of the kinematic chains of the system^20^. Thus, a system with a large number of actuators will be more complex than other systems with the same kinematics. Likewise, a system with a lower-order kinematic chain would be less complex than other systems with the same number of actuators. Thus, the mechanical complexity $C_M$ is defined as follows:

10 $$\begin{aligned} C_M=N_{A}\cdot N_{J} \end{aligned}$$

where $N_{A}$ is the number of actuators and $N_{J}$ is the number of joints. For example, the mechanical complexity of a CSTW mechanism^4^ with two actuators and two revolute joints is four.

#### Trajectory of CM

**Figure 9:**
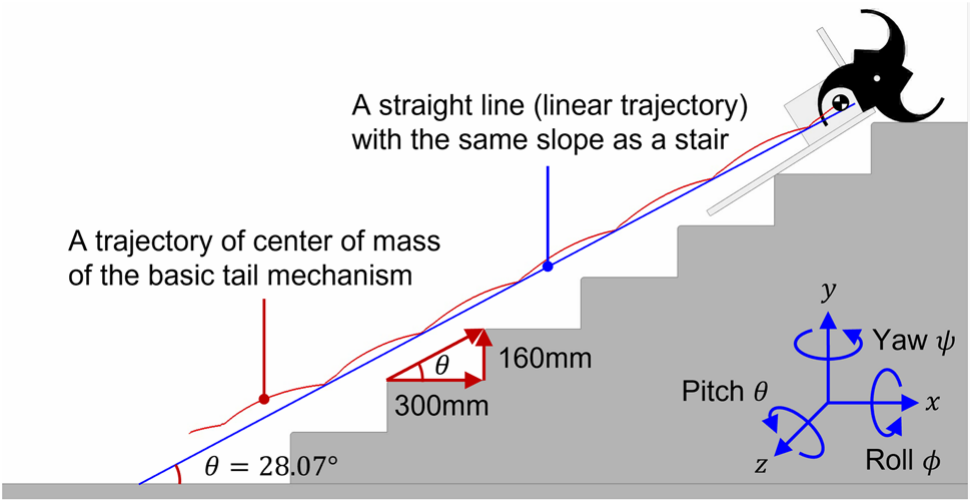
Trajectory of CM when climbing stairs in dynamic simulation. Software used for this figure: Microsoft PowerPoint Microsoft 365 MSO (Version 2209 Build 16.0.15629.20200) 64-Bit (https://www.microsoft.com/en-us/microsoft-365/powerpoint), Autodesk Inventor 2023.1.1 (https://www.autodesk.com/products/inventor/overview?term=1-YEAR &tab=subscription), RecurDyn V9R1 (9.1.23284.0.9108.3) (https://support.functionbay.com/en/page/single/2/recurdyn-overview).

The trajectory of the CM was selected to evaluate the stability and climbing performance of each mechanism. As shown in Fig. 9, the linearity of the trajectory of the CM was defined as the average and maximum values of the vertical (y-directional) displacement, with respect to the straight line (linear trajectory) having the same slope as the slope of the stairs. Smaller average and maximum values indicate better linearity of the CM trajectory of the mechanism, and by extension, stable stair-climbing. The maximum instantaneous value was critical for stable stair-climbing^7^.

Figure 9 shows the CM trajectory of the basic tail mechanism. The blue line was a straight line parallel to the slope of the stairs and the red curve represented the CM trajectory of the basic tail mechanism. For the basic tail mechanism, the average and maximum values of the vertical displacement with respect to the straight line were 14.73 mm and 27.31 mm, respectively.

#### Acceleration of CM

The mobile robot travels in various environments. When it travels under unfavorable conditions such as uneven road surfaces, stairs, protrusions, and slopes, its body shakes. The cause of such shaking is vibration or shock owing to the road surface environment, which makes it difficult for the robot to maintain a stable posture. Consequently, shaking degrades the stability of the robot. Other causes of shaking include the geometric characteristics of the road surface and robot driving mechanism, materials of the wheels and road surface, and driving speed^23,24^.

To evaluate the postural stability of mobile robots, the RMS acceleration of the CM was proposed. The method is an index that can quantitatively evaluate the random vibrations that occur in a robot during driving. The RMS acceleration value was used as the acceleration measurement result that was obtainable by calculating the RMS of the acceleration value in a predefined direction. The RMS acceleration is the total amount of energy physically transferred by random vibrations^25^.

In this study, the RMS accelerations were defined in two ways: translational and angular. The RMS translational acceleration was measured based on the translational acceleration in the direction of *x*, *y*, and *z*-coordinates of the CM of the robot during stairs climbing and can be derived as follows:

11 $$\begin{aligned} A_{T}=\sqrt{\frac{1}{N}\sum _{i=1}^{N}[(\ddot{x}_{i})^2+(\ddot{y}_{i})^2+(\ddot{z}_{i})^2]} \end{aligned}$$

where $\ddot{x}_{i}$, $\ddot{y}_{i}$, $\ddot{z}_{i}$ is the translational acceleration in the direction of *x*, *y*, *z*-coordinate of the CM at the *i*-th step in a simulation. *N* is the total number of simulation steps.

The RMS angular acceleration was measured based on the angular acceleration in the direction of the roll, pitch, and yaw of the CM of the robot during stairs climbing and could be derived as follows:

12 $$\begin{aligned} A_{A}=\sqrt{\frac{1}{N}\sum _{i=1}^{N}[(\ddot{\phi }_{i})^2+(\ddot{\theta }_{i})^2+(\ddot{\psi }_{i})^2]} \end{aligned}$$

where $\ddot{\phi }_{i}$, $\ddot{\theta }_{i}$, $\ddot{\psi }_{i}$ is the angular acceleration in the direction of roll, pitch, yaw of the CM at the *i*-th step in a simulation. where *N* is the total number of simulation steps.

If the RMS translational acceleration $A_{T}$ and the RMS angular acceleration $A_{A}$ were small, the shaking experienced owing to vibration or shock was minimal. Therefore, the mobile robot could be adjudged as stable.

#### Friction requirement (coefficient of friction)

The biggest impediments to stable stair-climbing are the friction and slip between the wheel and stairs. The driving wheel is prone to slipping when the frictional force acting between the wheel and stairs is less than the force generated by the driving torque of the wheel, which reduces the performance of the driving wheel and affects the stair-climbing performance of the robot. The slip of the driving wheel is related to the friction coefficient and normal force exerted by the wheel on the stairs and is determined by variables such as the payload of the robot, geometric characteristics of the driving mechanism and road surface shape, and the material of the wheel and stair surface^21,22^.

For friction, it is important to reduce the maximum required friction coefficient. When the actual friction coefficient between the wheel and the stair is smaller than the minimum required friction coefficient, slipping occurs and the robot cannot climb the stairs. In addition, the minimum required friction coefficient can be minimized by structural design to achieve high stair-climbing capability. Thus, the minimum required friction coefficient is frequently used to evaluate the stability of stair-climbing^26^.

In this study, the minimum required friction coefficient of the CSTW was determined to be the friction coefficient in the case where slipping does not occur in the CSTW to which each tail mechanism was applied through an iterative simulation process in the dynamic simulation.

#### Torque requirement (driving torque)

In terms of the torque requirement, the driving torque was used to evaluate the required torque of the motor for stable stair-climbing. Generally, the peak torque value was used to determine the required torque of the motor^21,22^. However, in the CSTW mechanism, the peak torque value of the driving torque occurred when the stopper of the CSTW made contact with the nosing; consequently, the effect of the tail mechanism on the peak torque value was minimal. Therefore, the RMS driving torque value was used to evaluate the degree of the overall influence of the tail mechanism on the driving torque.

The RMS driving torque was measured based on the driving torque of the CSTW during stairs climbing and was derived as follows:

13 $$\begin{aligned} T_{RMS}=\sqrt{\frac{1}{N}\sum _{i=1}^{N}({T}_{i})^2} \end{aligned}$$

where ${T}_{i}$ is the driving torque at the *i*-th step in a simulation, *N* is the total number of simulation steps.

#### Climbing speed

The stair-climbing speed is one of the most intuitive performance evaluation indices for different stair-climbing mechanisms. Each of the tail mechanisms has a different minimum required friction coefficient for stable stair climbing. It was, thus, imperative to determine a suitable approach to comparing them under the same conditions. Therefore, in the climbing-speed comparison, each mechanism was compared with the minimum required coefficient of friction.

The climbing speed is defined as the number of steps that can be climbed per second (step/s). In this study, it was obtained by measuring the time taken to climb six steps, as follows:

14 $$\begin{aligned} V_{C}=\frac{n}{t_{6}-t_{1}} \end{aligned}$$

where *n* is six steps, the number of steps climbed during the time between ${t}_{1}$ and ${t}_{6}$. ${t}_{6}$ is the time at which the stopper of the curved-spoke tri-wheel (CSTW) meets the edge of 6th step. ${t}_{1}$ is the time when the CSTW stopper meets the edge of the first step.

### Simulation environment

The proposed tail mechanisms were compared based on a dynamic simulation and evaluated using the performance indices introduced in “Performance indices”. The performance index values were obtained from the dynamic simulation results. Stair-climbing simulations of the five tail mechanisms were conducted using a commercial dynamic simulation tool (software), RecurDyn.

**Figure 10:**
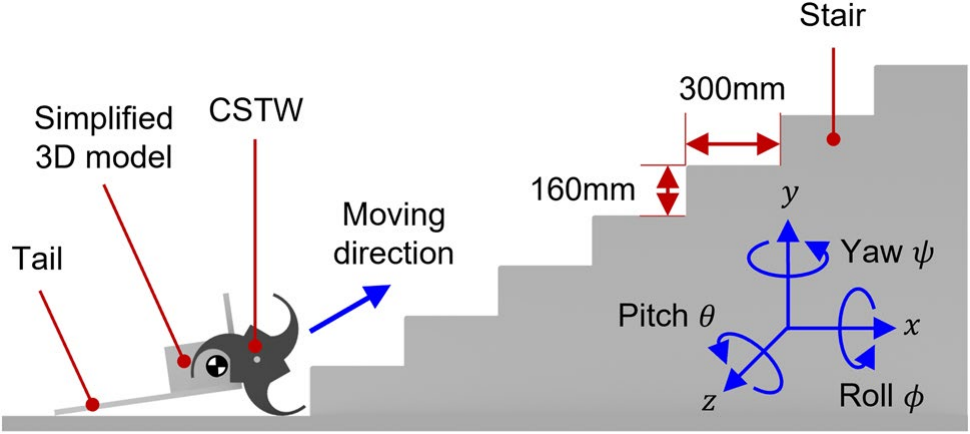
Simulation environment and condition. A tri-wheel-based robot with a tail mechanism climbed $300 \times 160$ mm stairs. Software used for this figure: Microsoft PowerPoint Microsoft 365 MSO (Version 2209 Build 16.0.15629.20200) 64-Bit (https://www.microsoft.com/en-us/microsoft-365/powerpoint), Autodesk Inventor 2023.1.1 (https://www.autodesk.com/products/inventor/overview?term=1-YEAR &tab=subscription).

The stair sizes are shown in Fig. 10, the stair riser height and nosing depth were assumed to be 160 mm and 300 mm, respectively, which is the most common stair size. A simplified 3D model of each tail mechanism was used for the simulation. Each model consisted of two CSTWs at the front and a different tail mechanism. The CSTW of the model was designed to fit the selected stair size. In the simulation, each climbing robot model had a control input that two CSTWs rotated at a speed of 20 rpm. Since the CSTW has a control input of a rotation speed of 20 rpm, the models were designed to climb one step per second.

The dynamic friction coefficient between the CSTW and stair surface was set from high (1.0, non-slip) to low to determine the minimum required friction coefficient for stable stair climbing. The minimum required friction coefficient of each tail mechanism was applied to compare the performance indices. The friction coefficient between the wheel of the tail mechanism and stair surface was assumed to be 1.0, a non-slip condition. In the case of the basic tail mechanism, friction occurred between the edge of the stairs and tail mechanism when the robot climbed the stairs and the coefficient of friction between the edge of the stairs and tail mechanism at that time was assumed to be 0.1. For the simulation, it was important to establish the contact conditions between the CSTW and stair surface and between the tail mechanism and stair surface. The stiffness and damping coefficients for the contact conditions were selected as 100,000 N/mm and 10 N/mm s, respectively. The simulation time and number of steps were seven seconds and 10,000 steps, respectively. The maximum time step, which was the upper-bound time-step size of the integrator during the dynamic analysis, was 1.e−003.

### Simulation result and discussion

#### Simulation result

To determine the suitable tail mechanism for the tri-wheel-based stair climbing robot among the five tail mechanisms, a dynamic simulation of stair climbing, was conducted to compare the performances of the five tail mechanisms: basic tail mechanism (conventional tail mechanism), curved linkage mechanism, tri-wheel mechanism, CPL-SD, and CPL-S. As discussed in the previous section, the stair-climbing simulation was performed using stairs with a size of $300 \times 160$ mm for each tail mechanism. A variety of performance indices, such as mechanical complexity, CM trajectory, CM acceleration, friction requirement (coefficient of friction), torque requirement (driving torque), and climbing speed, were utilized to evaluate and compare the tail mechanisms.

**Figure 11:**
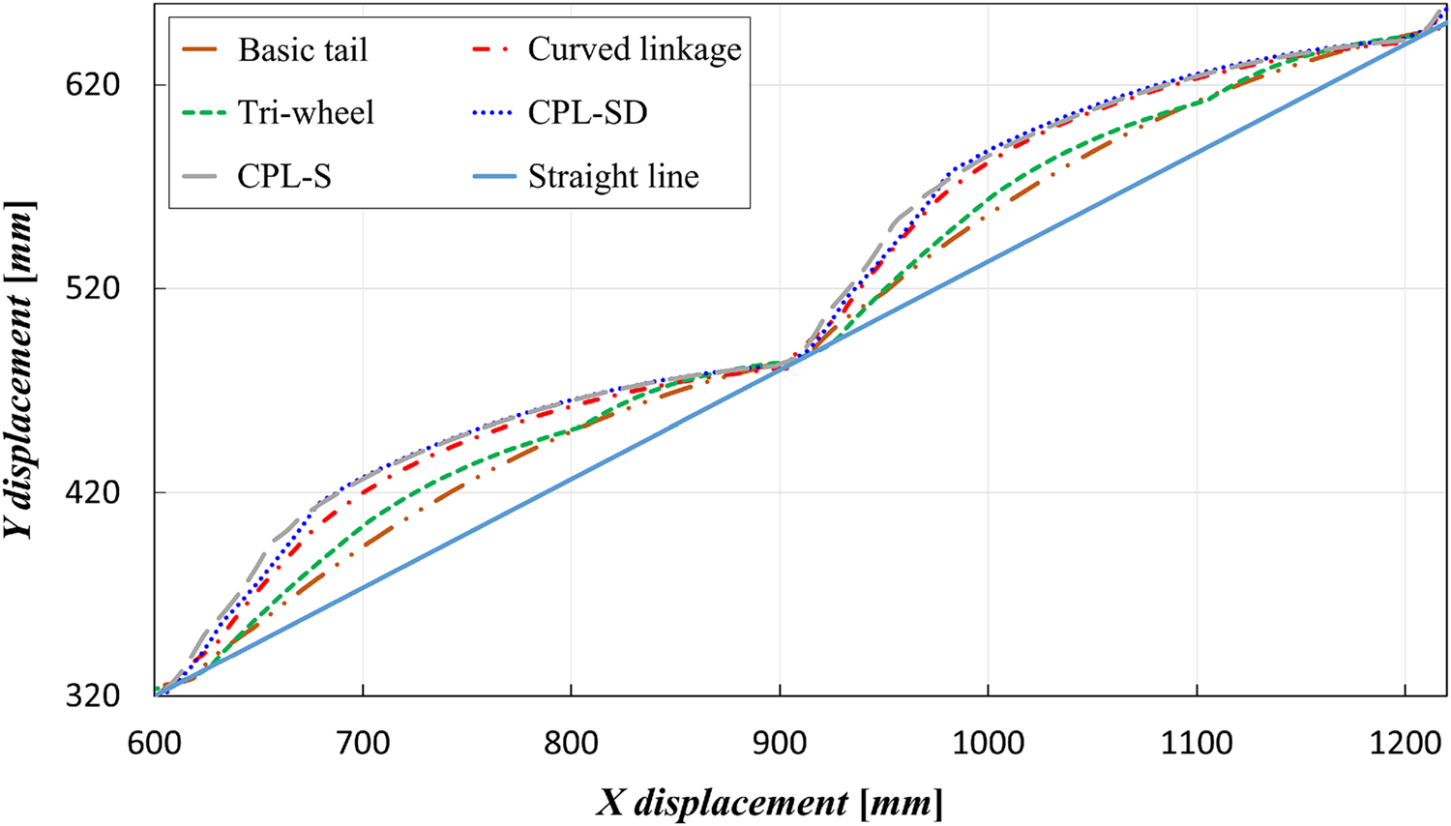
Simulation results for the trajectory of CM for stair climbing of five tail mechanisms.

**Figure 12:**
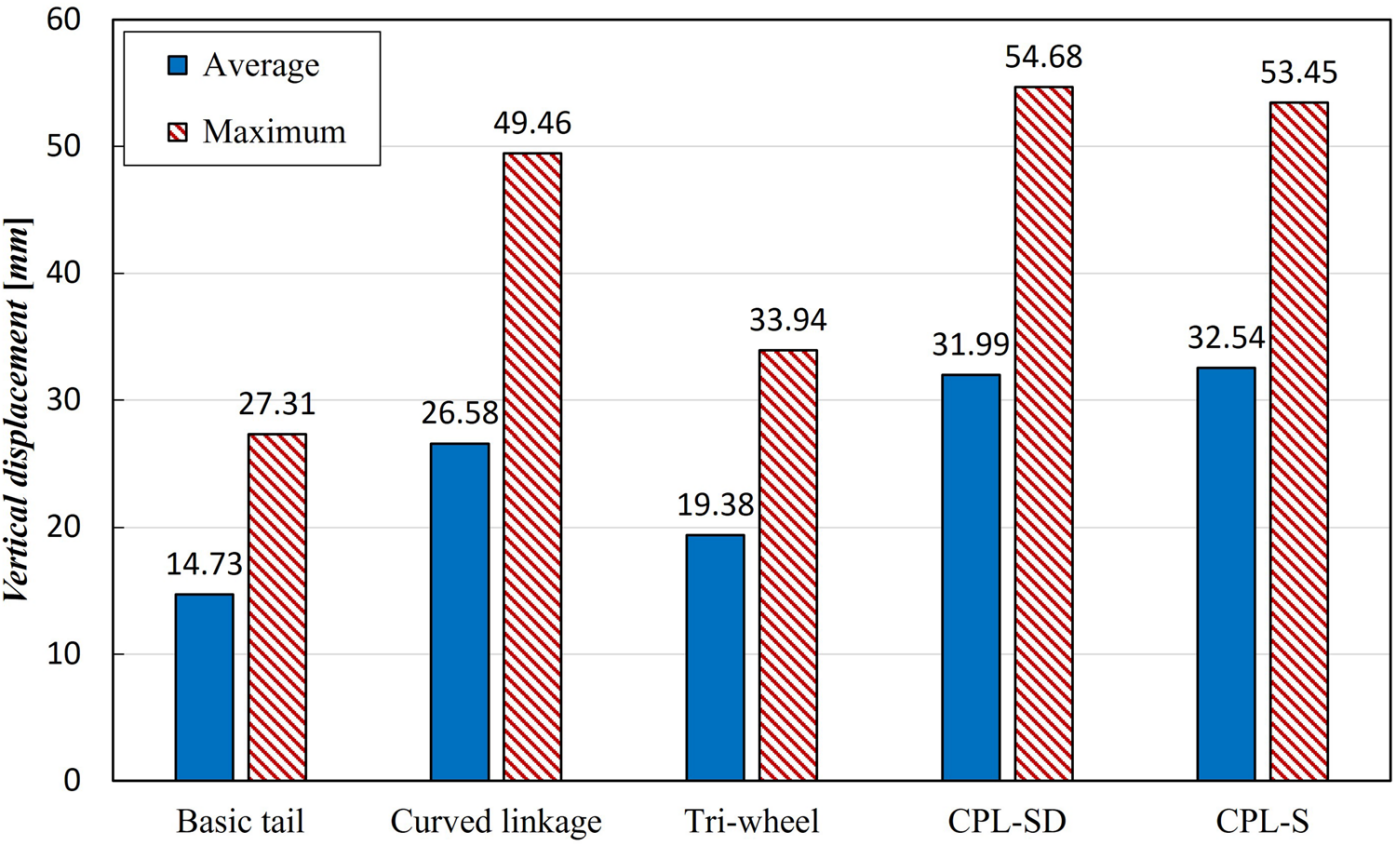
Linearity of the trajectory of CM of the five tail mechanisms: average and maximum value of the vertical displacement with respect to the straight line.

First, the simulation results for the CM trajectories of the five tail mechanisms that were used to evaluate the stability and climbing performance are shown in Fig. 11. The sky-blue line is a straight line indicating the slope of the stairs and the other five colored curves represent the CM trajectories of the five tail mechanisms. The simulation results for the linearity of the trajectory of CM introduced in “Trajectory of CM” are described in Fig. 12. For each of the tail mechanisms, the average and maximum values of the vertical displacement with respect to the straight line, which defined the linearity of the center-of-mass trajectory, are indicated.

As for the stair-climbing stability, it was observed that smaller average and maximum values corresponded to greater stability. The maximum instantaneous value was also an important indicator for the stability evaluation of a stair-overcoming robot. The basic tail mechanism had the smallest average and maximum values, 14.73 mm and 27.31 mm, respectively. The average and maximum values of the tri-wheel mechanism were 24.0% and 19.5% higher than the average and maximum values of the conventional tail mechanism, respectively. The rest of the tail mechanisms had larger averages and maximum values than those of the tri-wheel mechanism. Consequently, although it was not better than that of the basic tail mechanism, the tri-wheel mechanism exhibited the best linearity of the trajectory of the CM among the four proposed tail mechanisms (Curved linkage mechanism, Tri-wheel mechanism, CPL-SD, and CPL-S).

The stair-climbing performance of the tail mechanisms were evaluated based on the multiple performance indices mentioned in “Performance indices”: mechanical complexity, CM acceleration, friction requirement (coefficient of friction), torque requirement (driving torque), and climbing speed. The overall comparison outcomes of the five tail mechanisms for stair climbing are listed in Table 6.

**Table 6:** Comparison of performance indices of five tail mechanisms for stair climbing.

Mechanism	Basic tail mechanism	Curved linkage mechanism	Tri-wheel mechanism	CPL-SD	CPL-S
Simple model					
Mechanical complexity $C_M$	4	8	20	8	8
**Accl. of CM**					
RMS translational accel. $A_{T}$(mm/s$^{2}$)	$1.48\times 10^{4}$	$1.13\times 10^{4}$	$0.98\times 10^{4}$	$0.92\times 10^{4}$	$0.87\times 10^{4}$
RMS angular accel. $A_{A}$ (rad/s$^{2}$)	55.20	38.33	26.78	52.28	50.64
Friction requirement (coefficient of friction)	0.65	0.60	0.50	0.47	0.51
Torque requirement (RMS driving torque $T_{RMS}$) (N mm)	$1.83\times 10^{4}$	$1.25\times 10^{4}$	$1.25\times 10^{4}$	$1.01\times 10^{4}$	$0.96\times 10^{4}$
Climbing speed $V_{C}$ (step/s)	0.967	0.951	0.946	0.941	0.946

The mechanical complexity $C_M$, determined by the number of actuators $N_A$ and joints $N_J$ of the system, was calculated for each tail mechanism. Because the tail mechanisms are passive mechanisms without actuators, the number of actuators $N_A$ was two. Therefore, the mechanical complexity of each mechanism was mainly determined by the number of joints $N_J$. Table 6 shows the mechanical complexity of each tail mechanism. The mechanical complexity of the basic tail mechanism was four, which was the lowest among the five tail mechanisms. The mechanical complexities of the curved linkage mechanism, CPL-SD, and CPL-S were all equal to 8. The tri-wheel mechanism had the highest mechanical complexity of 20, which indicated that it was the most complex of the compared tail mechanisms.

**Figure 13:**
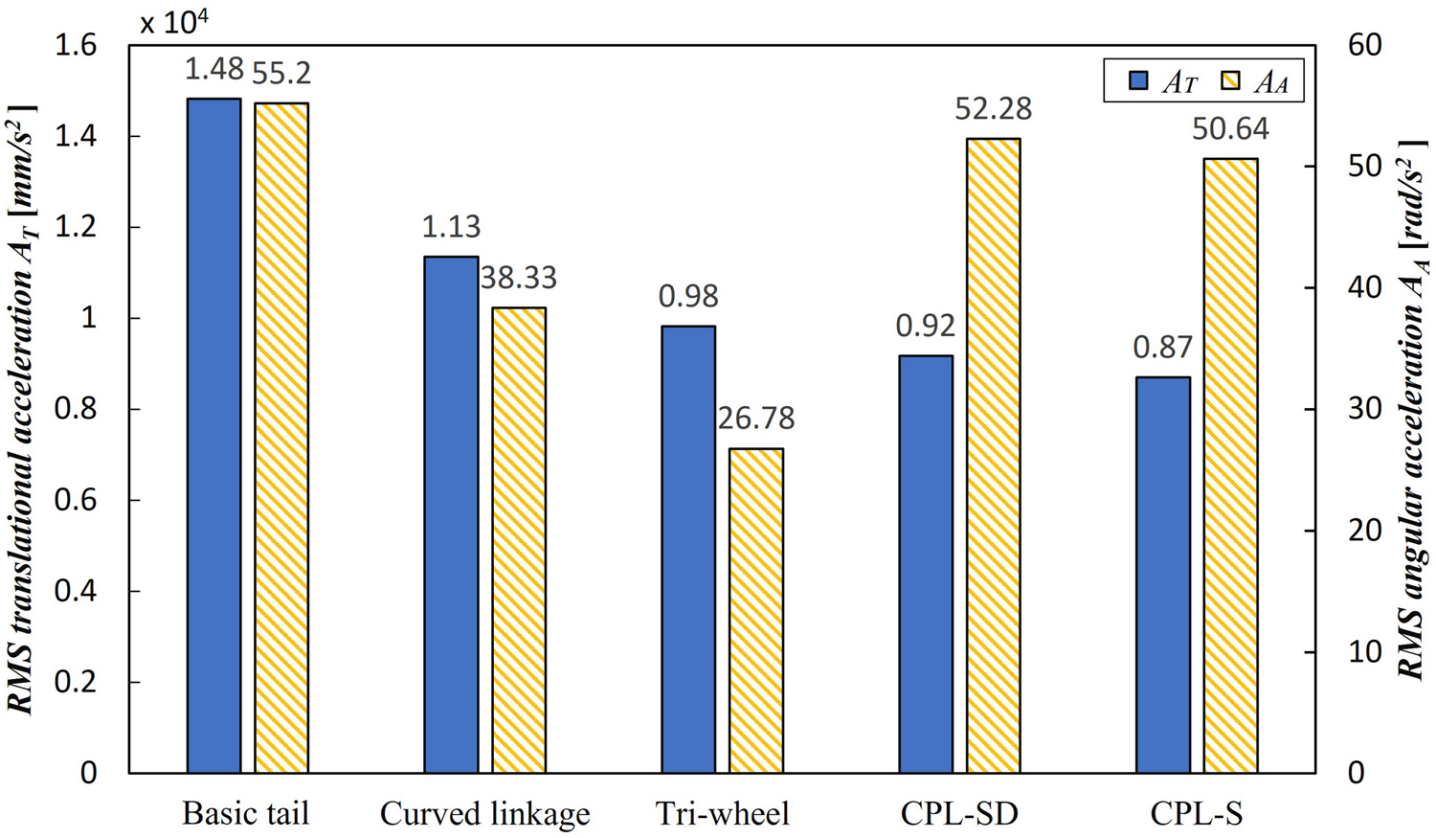
Comparison results of the RMS translational acceleration $A_T$ and the RMS angular acceleration $A_A$ of the five tail mechanisms.

The stability of the five tail mechanisms was evaluated based on the RMS acceleration of the CM. The RMS acceleration and acceleration measurement results obtained by calculating the RMS of the acceleration value in a predefined direction were of two types: the RMS translational acceleration $A_T$ and RMS angular acceleration $A_A$. A small RMS acceleration value indicated that the shaking caused by vibration or impact was minimal, which indicated that the robot had good stability. As can be seen in Fig. 13, the CPL-S had the least $A_T$, which was lower than the conventional tail mechanism by 41%. However, for $A_A$, the CPL-S exhibited a slight decrease of only 8%; thus, its value was similar to that of the basic tail mechanism. However, the tri-wheel mechanism had the least $A_A$ among the five tail mechanisms, which was 51% lower than that of the conventional tail mechanism. In addition, it had a significantly smaller $A_T$ value than that of the conventional tail mechanism, even though it was larger than that of the the CPL-S.

**Figure 14:**
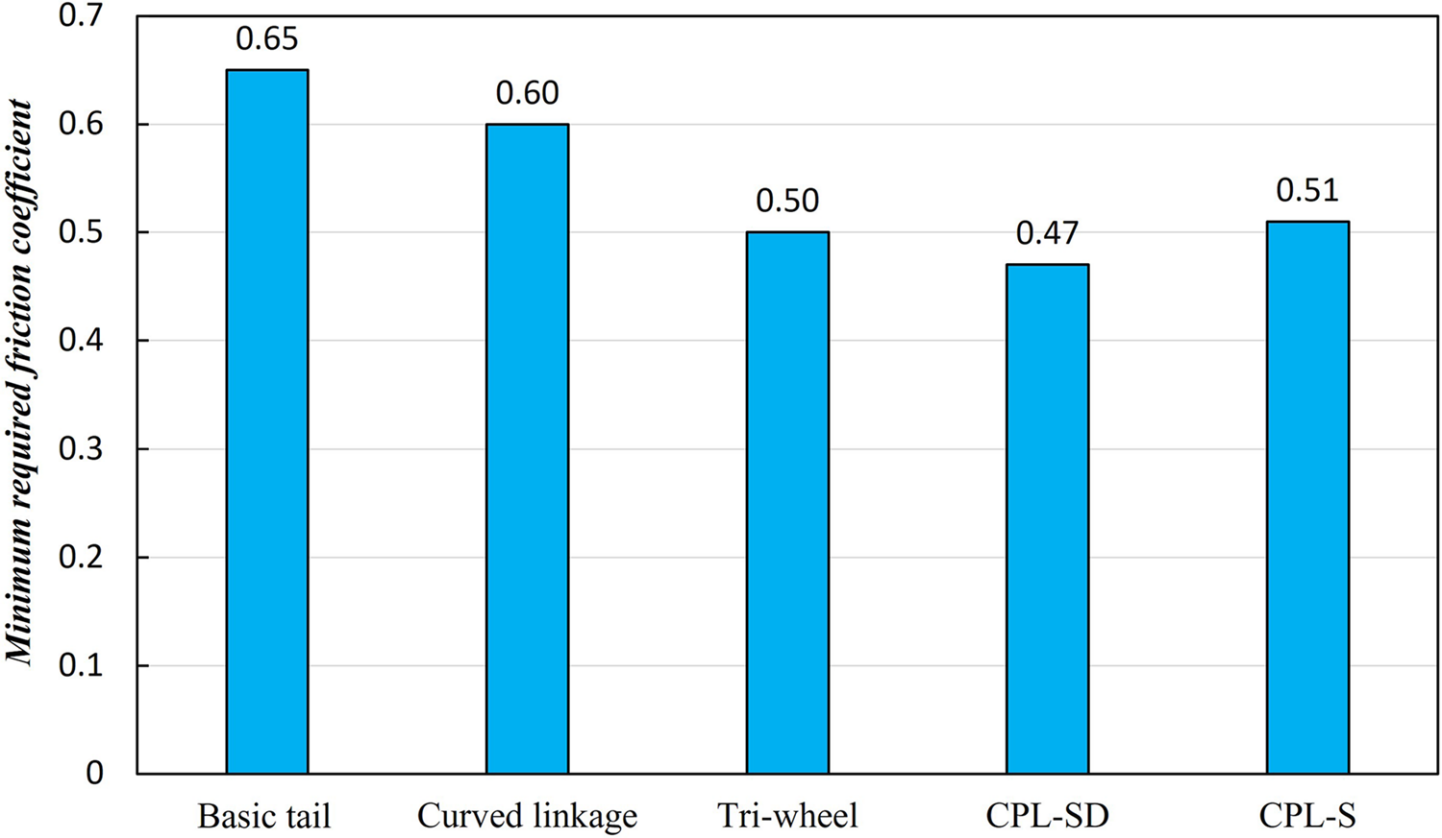
Comparison results of the minimum required friction coefficient for stable stair climbing of the five tail mechanisms.

To determine the friction requirement, each of the tail mechanisms was estimated based on the minimum required friction coefficient—one of the most important factors for stable stair climbing—between the CSTW and the stair surface. The coefficient of friction was closely related to the slip occurrence, which, if too frequent, degraded the driving wheel’s performance and affected the stair-climbing performance, as mentioned in “Friction requirement (coefficient of friction)”. The comparison results of the coefficient of friction for the five tail mechanisms are shown in Fig. 14. The mechanism with the lowest coefficient of friction was the CPL-SD, with a friction coefficient of 0.47, which was 28% lower than that of a conventional tail mechanism.

**Figure 15:**
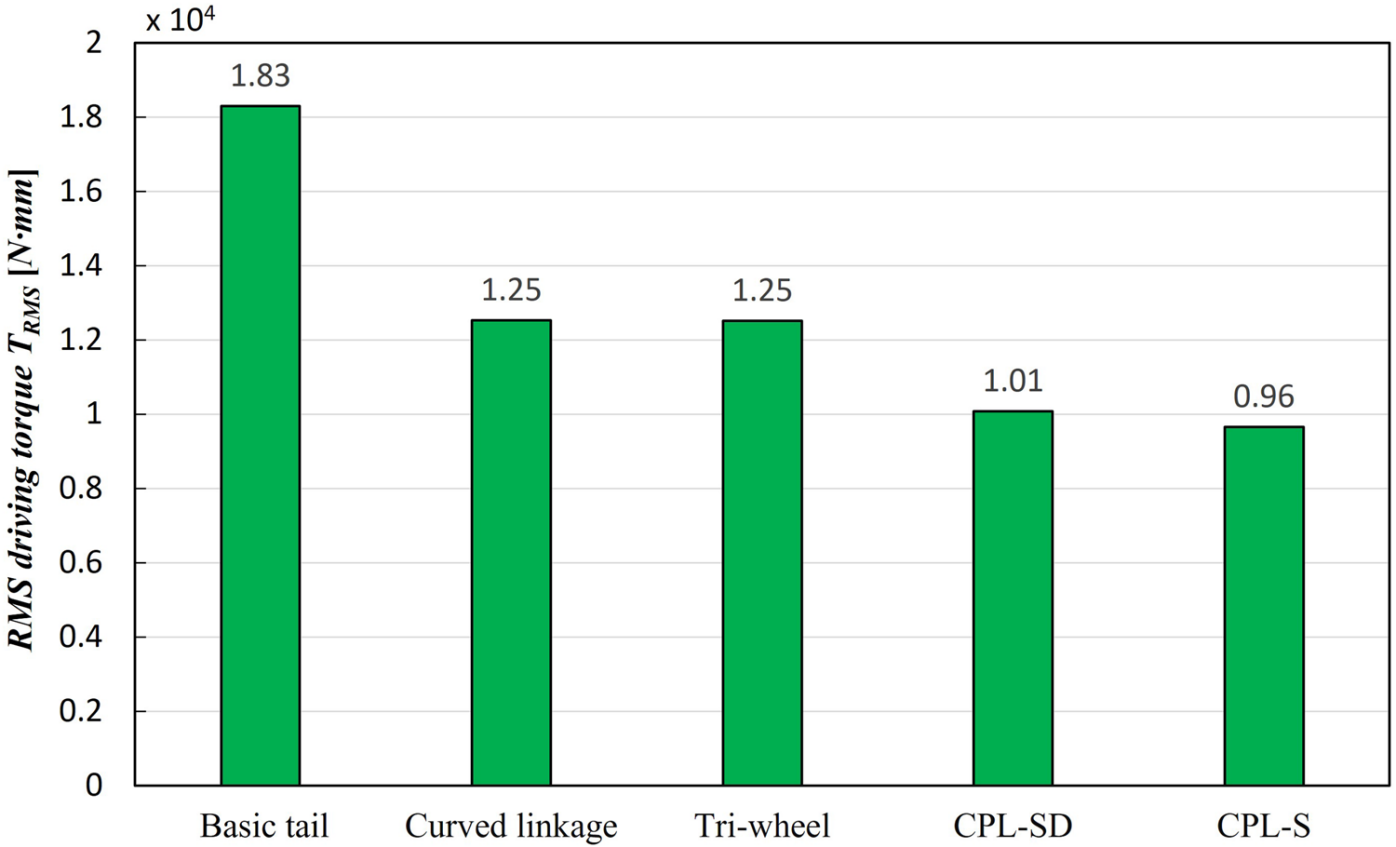
Comparison results of the RMS driving torque $T_{RMS}$ of the five tail mechanisms.

The RMS driving torque was also utilized to estimate the required torque of the motor for stable stair-climbing. In the case of the RMS driving torque $T_{RMS}$, as shown in Fig. 15, the CPL-S exhibited the largest decrease, compared to the conventional tail mechanism, and had the smallest RMS driving torque of the five tail mechanisms. The decrease was 28% lower than that of the conventional tail mechanism. Although the values of the remaining tail mechanisms were larger than that of the CPS-S, they also showed significantly reduced values compared with the conventional tail mechanism.

As abovementioned, the climbing speed is the simplest yet most intuitive evaluation index for comparing the performance of different stair-climbing mechanisms. It is defined as (step/s) and the number of steps a mechanism can climb per second. The comparison results of the climbing speed of the tail mechanisms can be found in Table 6. Although the basic tail mechanism, the conventional tail mechanism, had a slightly faster climbing speed than the other tail mechanisms, the mechanisms exhibited similar climbing speeds. Hence, it was confirmed that the proposed tail mechanisms did not reduce the climbing speed.

Comparative analysis of tail mechanisms through various performance indicators was conducted. For a more clear and distinct comparative analysis, a single performance indicator was derived through min-max normalization (rescaling) and weight assignment of each performance indicator as follows.

15 $$\begin{aligned} {x}^\prime = \frac{x-min(x)}{max(x)-min(x)} \end{aligned}$$

where *x* is an original value of each performance indicator and ${x}^\prime$ is the normalized value of each performance indicator.

**Table 7:** Weight assignment of each performance indicator.

Performance indices	Average of lin. of CM traj.	Maximum of lin. of CM traj.	$C_M$	$A_T$	$A_A$	Min. rqd. friction coefficient	$T_{RMS}$	1-$V_{C}$
Weight	0.05	0.10	0.05	0.15	0.15	0.30	0.15	0.05

**Figure 16:**
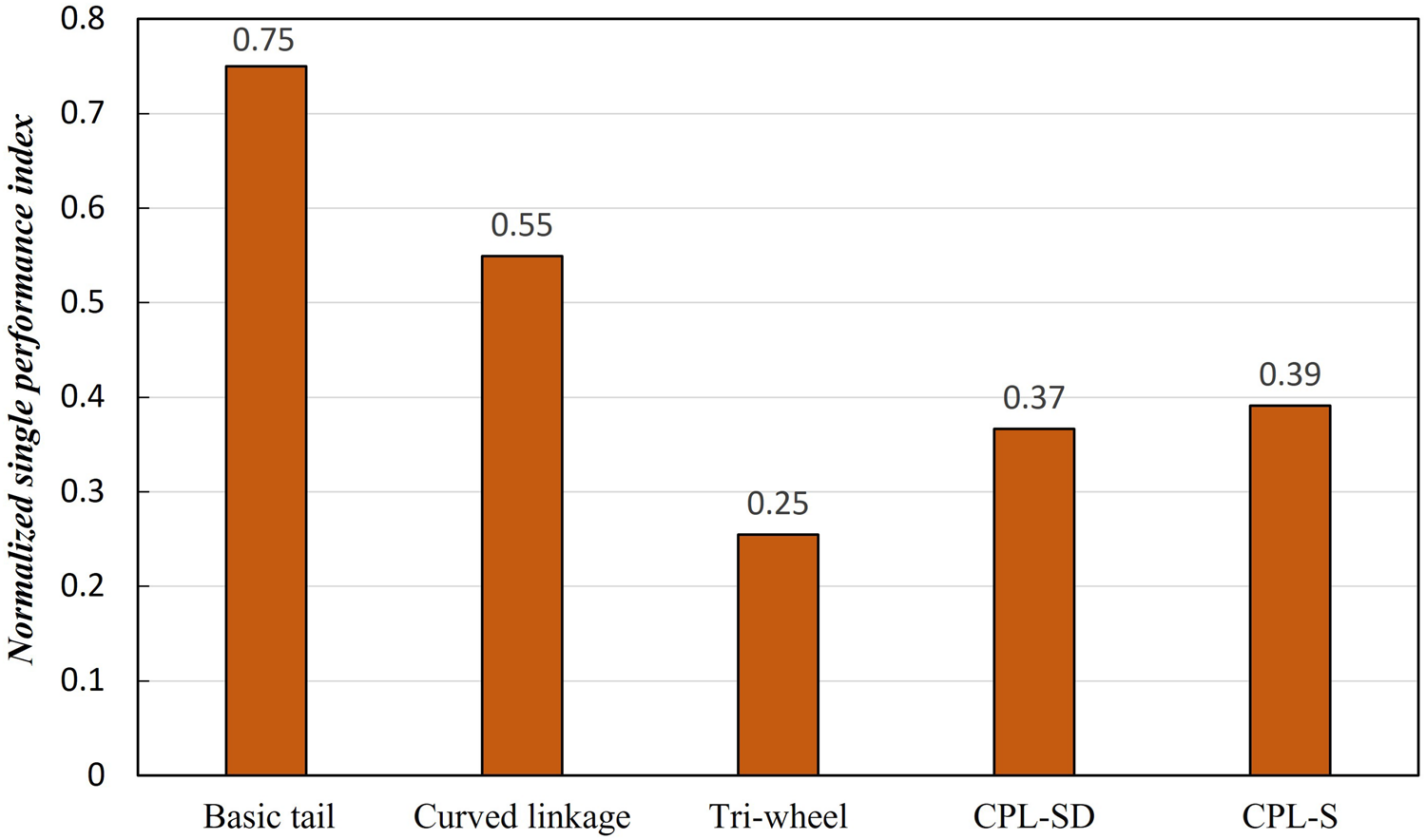
Comparison results of the normalized single performance index of the five tail mechanisms.

The eight performance indicators used in the comparative analysis (average and maximum value of the linearity of the trajectory of CM, $C_M$, $A_T$, $A_A$, minimum required friction coefficient, $T_{RMS}$, and $1-{V_{C}}$) were given different weights as shown in Table 7, and the normalized single performance indicators were derived by multiplying the weights given to the normalized values of each performance indicator. Figure 16 shows the comparison results of the normalized single performance index of the five tail mechanisms. The performance indices mean that the smaller the value, the better the performance, so a single normalized performance index also indicates that the smaller the value, the better the performance. The tri-wheel mechanism was found to have the smallest normalized single performance index value among the five tail mechanisms.

## Discussion

Through the performance comparison evaluation of the five tail mechanisms in “Result”, the tail mechanism that exhibited excellent stability and stair-climbing performance and solved the problems dogging tri-wheel-based stair-climbing robots arising from impact during locomotion and friction with the nosing was determined.

Although LEVO’s tail mechanism, the conventional tail mechanism, had the least mechanical complexity, it also yielded overall poor results in terms of the CM acceleration, driving torque, and minimum required friction coefficient. Specifically, in the case of the minimum required friction coefficient, the other tail mechanisms were found to be able to climb stairs even if they slipped at a friction coefficient that was slightly lower than the minimum required friction coefficient. However, LEVO’s tail mechanism was found to be unsuitable for climbing stairs at all owing to frequent slips in these conditions.

The three mechanisms, curved linkage mechanism, CPL-SD, and CPL-S, had similar forms and the same mechanical complexity; however, they differed based on other performance indicators, such as CM acceleration, minimum required friction coefficient, and driving torque. This difference was mainly attributable to the presence or absence of compliance with the tail mechanism. With compliance via spring, the CPL-SD and CPL-S exhibited small RMS translational acceleration values, minimum required friction coefficients, and RMS driving torque values, compared to the curved linkage mechanism.

Finally, although the tri-wheel mechanism had the highest mechanical complexity among the tail mechanisms, it exhibited excellent results overall based on the other performance indices. Therefore, although it did not exhibit the best performance improvement in terms of all the performance indicators, it showed better overall good performance improvement based on all the performance indicators, compared to the conventional tail mechanism, and was therefore adjudged the best among the proposed tail mechanisms.

## Experimental verification and results

### Experiment condition and prototype robot

For an experimental verification of the tail mechanism suggested in this study, a stair-climbing experiment was conducted using the prototype of the tail mechanism. As a verification experiment, it was decided to compare the performance of the basic tail mechanism and the tri-wheel mechanism, which showed the best performance improvements among the proposed tail mechanisms, in the experiment using measurable performance indices among the performance indices adopted in the comparison with dynamic simulation.

**Figure 17:**
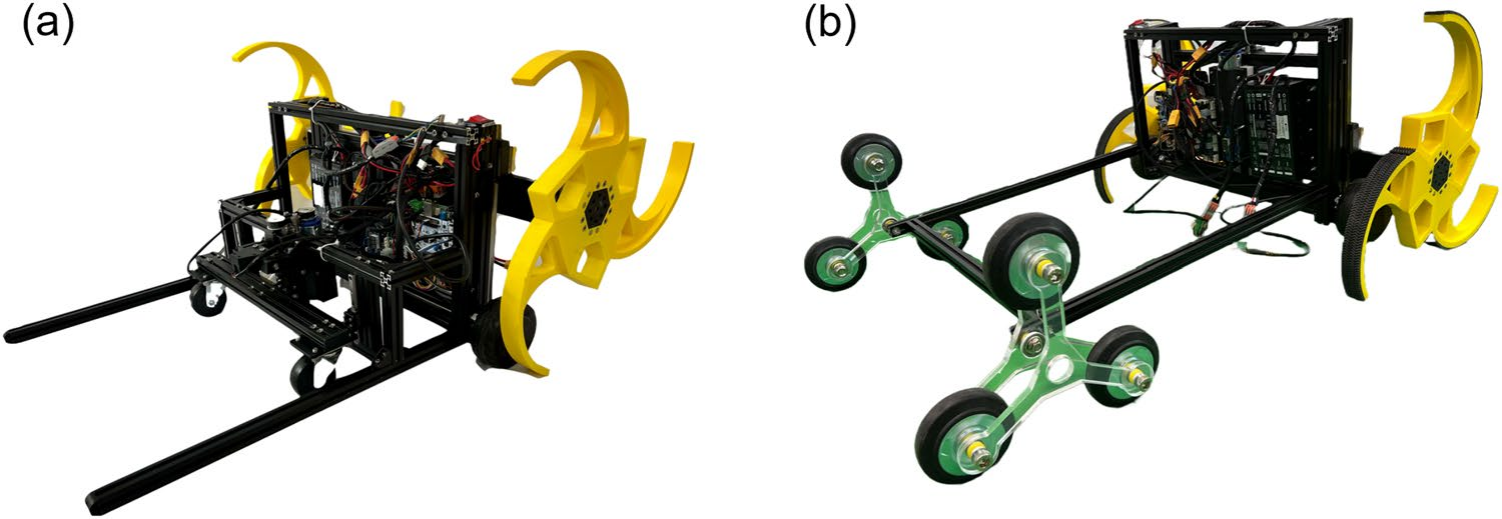
Prototype robots for the experiment: (**a**) basic tail mechanism, (**b**) tri-wheel mechanism. Software used for this figure: Microsoft PowerPoint Microsoft 365 MSO (Version 2209 Build 16.0.15629.20200) 64-Bit (https://www.microsoft.com/en-us/microsoft-365/powerpoint).

Two prototypes—the basic tail mechanism, the tri-wheel mechanism—were designed and created for experimental verification. Figure 17 shows the two prototypes used in the stair-climbing experiments. These prototypes were built based on the selected cases of the design parameters in “Tail mechanism design”, as shown in Table 8. The dimensions of the two prototypes use the values of each of the aforementioned design parameters, and the width in all cases is 700 mm. The CSTW was fabricated by 3D printing based on the design parameters of the CSTW described in the preceding research^4^ to fit the stair size of $300 \times 160$ mm. As the driving motor of the CSTW, servo motors (PH54-200-S500-R, ROBOTIS), including the encoder and controller, were used. The body and the tail mechanism of the prototype consisted of aluminum frames. The spokes of the tri-wheel mechanism were manufactured by cutting acrylic plates using a laser cutter according to design parameter $l_2$. The wheels of the tri-wheel mechanism were commercial wheels of a size that fit the value of the design parameter $d_w$.

**Table 8:** Design parameters of the two prototypes built for the experimental verification.

Tail mechanism	Design parameter				
	$l_1$	$l_2$	$\theta$	$d_w$	*h*
Basic tail mechanism	550	–			
Tri-wheel mechanism (case 2)	550	115	120	100	64

**Figure 18:**
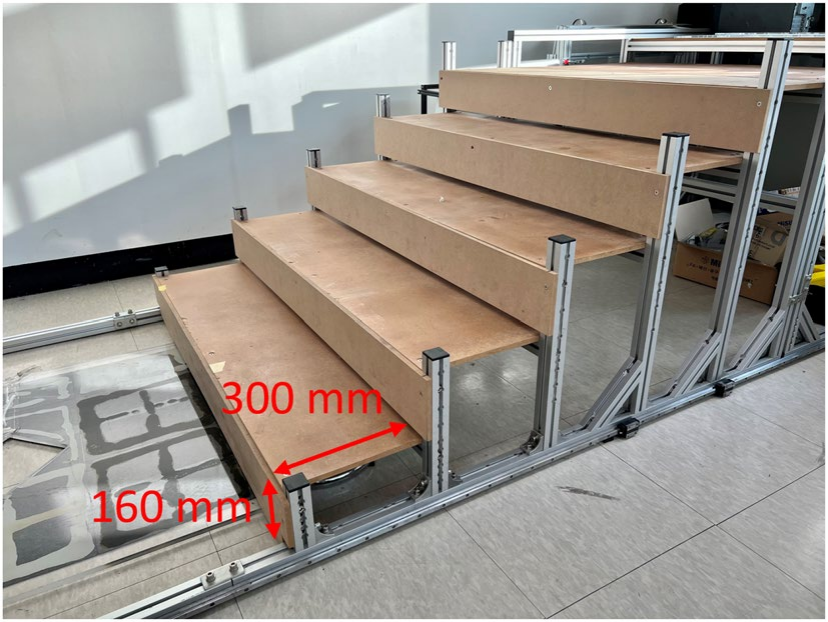
Test-bed used in the stair-climbing experiment: Stairs of $300 \times 160$ mm.

As shown in Fig. 18, stairs of $300 \times 160$ mm were used for the stair-climbing experiments of the tail mechanism prototypes. In the stair-climbing experiments, the rotation speed of the CSTW was set to 20 rpm.

### Experimental results

**Figure 19:**
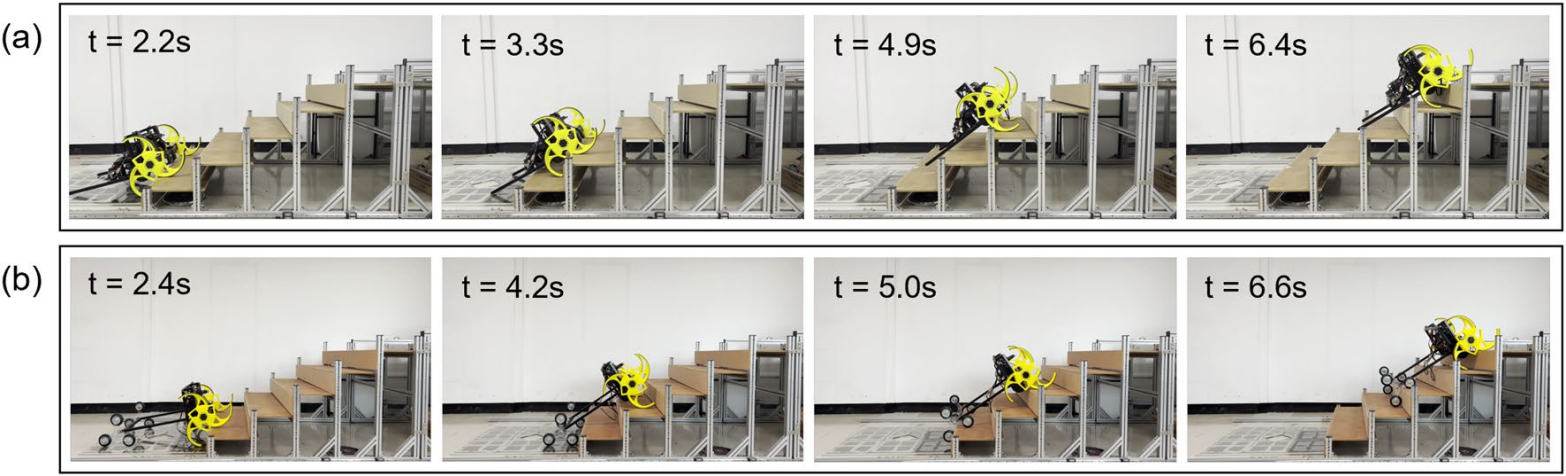
Experimental results of two tail mechanisms: (**a**) basic tail mechanism, (**b**) tri-wheel mechanism.

As shown in Fig. 19, stair-climbing experiments of the two tail mechanisms (the basic tail mechanism and the tri-wheel mechanism) were conducted. In the experiment, measurable data of the performance indices, such as the translational acceleration in the direction of *x*, *y*, *z*-coordinate of the CM, the angular acceleration in the direction of roll, pitch, yaw of the CM, the driving torque and the climbing speed, were measured and analyzed to validate the performance improvement of the proposed tail mechanism. Based on the measured data, the RMS translational acceleration $A_T$, the RMS angular acceleration $A_A$, the RMS driving torque $T_{RMS}$, and the climbing speed $V_{C}$ of two tail mechanisms were calculated using the definitions introduced in “Performance indices”.

Table 9 summarizes the comparison results of the performance indices (the RMS translational acceleration $A_T$, the RMS angular acceleration $A_A$, the RMS driving torque $T_{RMS}$, and the climbing speed $V_{C}$) between the basic tail mechanism and the tri-wheel mechanism obtained through simulations and experiments. According to the results of the experimental verification, it can be concluded that the comparative analysis results based on the simulation is reliable since the performance indices between the simulation and the experiment of the tail mechanisms are similar. Despite there is a small value difference between the simulation and experiment, it is speculated that the difference in these values is due to the model not perfectly matching the simulation and the experiment.

**Table 9:** Comparison results of the performance indices between the basic tail mechanism and the tri-wheel mechanism in the simulations and the experiments.

Mechanism	Simulation		Experiment	
	Basic tail mechanism	Tri-wheel mechanism	Basic tail mechanism	Tri-wheel mechanism
**Accl. of CM**				
RMS translational accel. $A_T$ (mm/s$^2$)	$1.48\times 10^{4}$	$0.98\times 10^{4}$	$1.12\times 10^{4}$	$0.74\times 10^{4}$
RMS angular accel. $A_A$ (rad/s$^2$)	55.20	26.78	58.50	28.53
Torque requirement (RMS driving torque $T_{RMS}$) (N mm)	$1.83\times 10^{4}$	$1.25\times 10^{4}$	$1.82\times 10^{4}$	$1.49\times 10^{4}$
Climbing speed $V_{C}$ (step/s)	0.967	0.946	0.959	0.962

## Conclusion

In this study, several tail mechanisms were proposed for tri-wheel-based stair-climbing robots. The tail mechanisms were designed such as to improve the stability and stair-climbing performance of tri-wheel-based stair-climbing robots when climbing stairs and to solve the problems faced by these robots during stairs climbing. To evaluate the performance improvement, the tail mechanisms were compared and analyzed through dynamic simulations based on various performance indices. Comparative analysis through evaluation indicators confirmed that the tri-wheel tail mechanism was the best of all the tail mechanisms and that the proposed mechanism improved on the conventional tail mechanism most significantly. In addition, experimental verification was performed, and it confirmed the reliability of the comparative analysis results based on the simulation. In conclusion, applying these tail mechanisms will not only ensure that the tri-wheel-based stair-climbing robots have excellent stair climbing stability and stair-climbing performance but will also solve problems that tend to affect stair climbing.

In future work, a robust optimal design of the tri-wheel tail mechanism is required to maximize the performance improvement of the stair-climbing robot through the tri-wheel tail mechanism, which showed the best performance in this study, and to cope with stairs of various sizes.

## Data Availability

Due to space limitation, this paper only shows data results processed from raw data using the definition of the introduced performance indices. The raw data generated during and/or analyzed during the current study are available from the corresponding author on reasonable request.
